# P2X7R/NLRP3 signaling pathway-mediated pyroptosis and neuroinflammation contributed to cognitive impairment in a mouse model of migraine

**DOI:** 10.1186/s10194-022-01442-8

**Published:** 2022-07-02

**Authors:** Yajuan Wang, Zhengming Shan, Lily Zhang, Shanghua Fan, Yanjie Zhou, Luyu Hu, Yue Wang, Weidong Li, Zheman Xiao

**Affiliations:** Department of Neurology, Renmin Hospital of Wuhan University, 99 Zhang Zhidong Road, Wuchang District, Wuhan, 430060 Hubei Province China

**Keywords:** Migraine, Cognitive impairment, P2X7 receptor, NLRP3 inflammasome, Pyroptosis, Apoptosis, Neuroinflammation

## Abstract

Migraine is the second most common form of headache disorder and the second leading cause of disability worldwide. Cognitive symptoms ranked second resulting in migraine-related disability, after pain. P2X7 receptor (P2X7R) was recently shown to be involved in hyperalgesia in migraine. However, the role of P2X7R in migraine-related cognitive impairment is still ill-defined. The aim of this study was to explore the molecular mechanisms underlying migraine-related cognitive impairment and the role of P2X7R in it. Here we used a well-established mouse model of migraine that triggered migraine attacks by application of inflammatory soup (IS) to the dura. Our results showed that repeated dural IS stimulation triggered upregulation of P2X7R, activation of NLRP3 inflammasome, release of proinflammatory cytokines (IL-1β and IL-18) and activation of pyroptotic cell death pathway. Gliosis (microgliosis and astrogliosis), neuronal loss and cognitive impairment also occurred in the IS-induced migraine model. No significant apoptosis or whiter matter damage was observed following IS-induced migraine attacks. These pathological changes occurred mainly in the cerebral cortex and to a less extent in the hippocampus, all of which can be prevented by pretreatment with a specific P2X7R antagonist Brilliant Blue G (BBG). Moreover, BBG can alleviate cognitive impairment following dural IS stimulation. These results identified P2X7R as a key contributor to migraine-related cognitive impairment and may represent a potential therapeutic target for mitigating cognitive impairment in migraine.

**Supplementary Information:**

The online version contains supplementary material available at 10.1186/s10194-022-01442-8.

## Introduction

Migraine is the second most common form of headache disorder [1], and is the second leading cause of disability worldwide [2]. Cognitive symptoms, particularly impairment in visuospatial abilities, processing speed, attention, memory and executive functions [3–6], ranked second resulting in migraine-related disability, after pain [7]. Thus, there is an urgent need to identify the mechanisms and effective interventions for migraine-related cognitive impairment.

P2X7 receptor (P2X7R), a purinergic receptor family member, was demonstrated to be involved in the pathogenesis and progression of migraine [8]. P2X7R can be activated by extracellular ATP [9], which was significantly elevated in animal models of migraine and patients with migraine [10, 11]. The upregulation of P2X7R in trigeminal nucleus caudalis (TNC) in a mouse model of migraine provided more direct evidence for its involvement in migraine [12]. Genetical depletion or pharmacological inhibition of P2X7R can alleviate nitroglycerin (NTG)-induced mechanical and thermal hyperalgesia in a mouse model of migraine [12, 13]. Moreover, P2X7R was recently found to facilitate cortical spreading depression (CSD) propagation [14, 15], which is known to be a trigger for migraine attacks.

Despite that the role of P2X7R in migraine pathogenesis has been proved, its relationship with migraine-related cognitive impairment is not well characterized. The activation of P2X7R can promote the formation of NLRP3 inflammasome [16, 17], which is an intracellular multimeric protein complex that initiates inflammatory response and cell death (pyroptosis and apoptosis) by activating its effector caspase-1 [18–20]. The active caspase-1 facilitates the production of proinflammatory cytokines IL-1β and IL-18 [21–23], and activates pyroptosis-related protein Gasdermin D (GSDMD) and apoptosis-related protein caspase-3 [24–26]. This may lead to gliosis, neuronal loss and white matter damage, consequently leading to cognitive impairment [27]. The P2X7R-NLRP3 route has been demonstrated to be involved in cognitive impairment in several neurological diseases such as Alzheimer's disease (AD), vascular dementia (VaD) and diabetes [28–30].

Evidence from clinical studies showed that cognitive symptoms of migraineurs may be associated with the structural and functional abnormalities in the cerebral cortex, hippocampus and white matter [5, 31, 32]. It has been shown that inflammasome activation can mediate cognitive decline in a mouse model of VaD by inducing gliosis, neuronal loss in the cerebral cortex and hippocampus, and white matter damage [27]. Since multiple inflammasome-associated proteins including NLRP3, IL-18 and IL-1β, have been shown to be upregulated in migraine [33, 34], we hypothesized that P2X7R might participate in the development of cognitive dysfunction in migraine by activating NLRP3 inflammasome signaling pathway, which may induce the gliosis and neuronal loss in the cerebral cortex and hippocampus, and impairment of white matter, ultimately resulting in cognitive impairment.

Here we used a well-established mouse model of migraine that triggered migraine attacks by application of inflammatory soup (IS) to the dura [35, 36]. We evaluated the effects of repeated dural IS stimulation on NLRP3 inflammasome signaling pathway, cognition-related pathological changes (gliosis, neuronal loss and white matter damage) and cognitive behavior. We determined whether these possible pathological changes and cognitive decline were mediated by P2X7R using a specific P2X7R antagonist, Brilliant Blue G (BBG).

## Materials and methods

### Animals

**Fig. 1:**
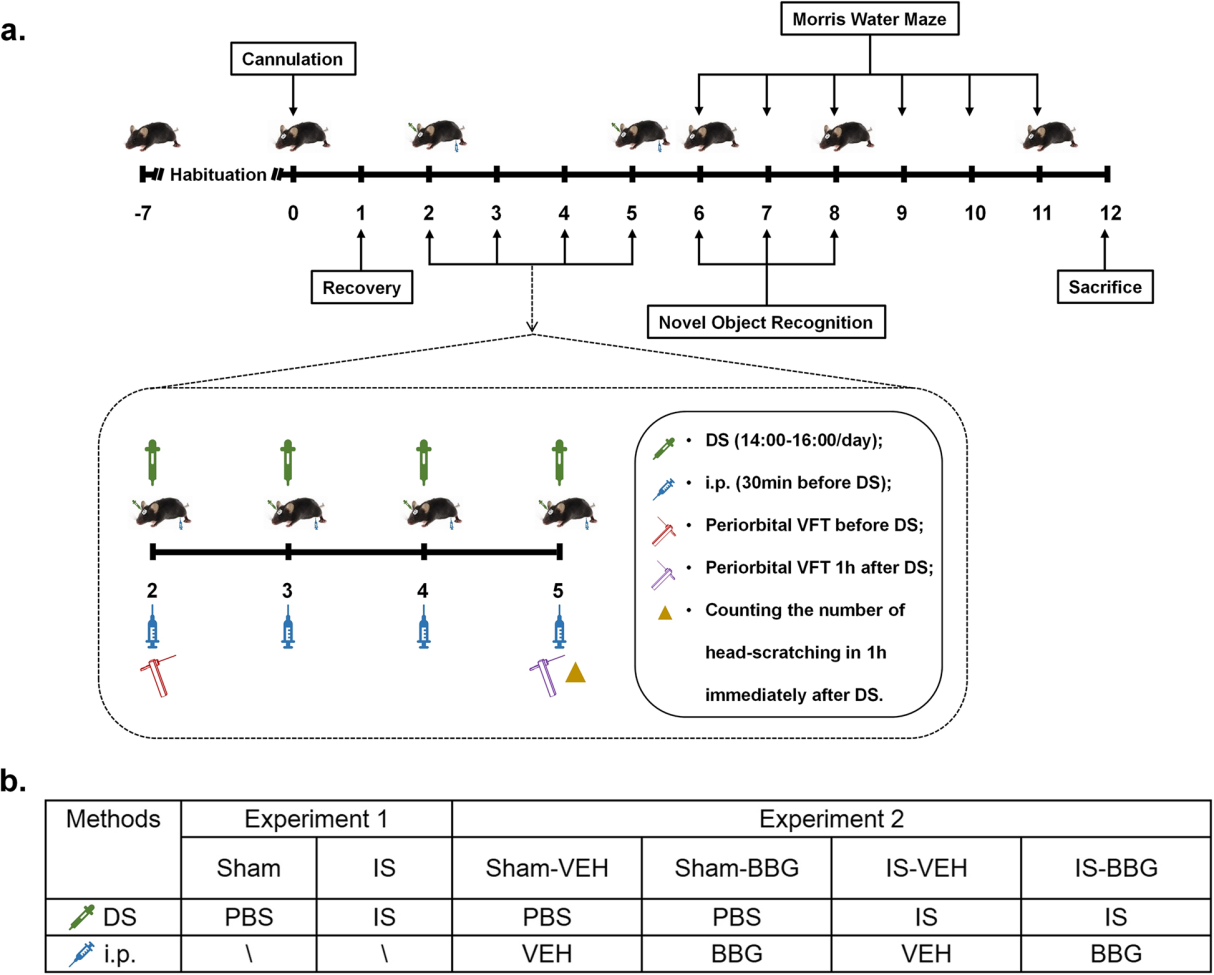
The experimental protocol of the study. (**a**) Schematic timeline diagram of drug treatment and behavioral assessment in mice. (**b**) Details of the dosing regimen for each group of mice. IS, 20μL/day for 4 days, dural injection; PBS, 20μL/day for 4 days, dural injection; BBG, a specific P2X7R antagonist, 50 mg/kg/day for 4 days, intraperitoneal injection; VEH, 0.9% saline, the same volume as BBG, intraperitoneal injection. Abbreviations: DS, dural stimulation; i.p., intraperitoneal injection; VFT, von Frey’s test; IS, inflammatory soup; PBS, phosphate buffer saline; BBG, brilliant blue G; VEH, vehicle

Seven to eight weeks old male C57BL/6 mice weighing 22-28 g were used in the study. All mice were purchased from the Charles River Laboratories. Mice were housed under specific pathogen-free (SPF) conditions in the Animal Experiment Center of Renmin Hospital of Wuhan University, where food and water were provided ad libitum with temperature 22 ± 2℃, humidity 60 ± 5% and 12-h light/dark cycle. All efforts were taken to minimize animal suffering and reduce the number of animals used. Experimental procedures were approved by the Institutional Animal Care and Use Committee (IACUC) of Renmin Hospital of Wuhan University, with the IACUC issue No. WDRM animal (welfare) 20,201,101. The experiments were reported in accordance with the ARRIVE (Animal Research: Reporting In Vivo Experiments) guidelines. In experiment 1, the mice were randomly assigned to each group: (1) sham, and (2) IS. In experiment 2, the mice were randomly assigned to the following groups: (1) sham-vehicle (VEH, 0.9% saline), (2) sham-BBG (a P2X7R antagonist), (3) IS-VEH, and (4) IS-BBG. Detailed grouping is shown in Fig. 1b. In all experiments, investigators were blind to animal grouping. The mice were acclimated for one week prior to experiment.

### Cannula implantation

Mice were anesthetized initially by 3% isoflurane via anesthetic-specific vaporizers; once the paw pinch reflex of mice disappeared, the concentration of isoflurane was lowered to 1.5% throughout the surgery. The scalp was incised longitudinally and the connective tissues were removed to expose the bregma. A small skull hole (diameter 1 mm, 1 mm after and 1 mm lateral to the bregma) was drilled penetrating through the skull with the dura intact using a skull drill (Reward, 87,001). A plastic cylindrical container (diameter 5 mm, height 2 mm) without a bottom lid was used as a guide cannula. The top of the container has two holes (diameter 1 mm) to facilitate the insertion of the microinjector. The guide cannula was implanted over the previously drilled skull hole and sealed into place with glue. The skin incision was then closed with surgical suture. After surgery, topical erythromycin was administered to each mouse to prevent infection. Mice were returned to their home cage and allowed to recover for 24 h. The detailed procedures have been reported previously [35].

### Drug administration

#### Dural injection

Mice in the IS, IS-VEH and IS-BBG groups received dural injection of IS to induce migraine-like headache attacks. IS was prepared freshly each day with PBS containing 0.1 mM prostaglandin E2, 1 mM serotonin, 1 mM histamine and 1 mM bradykinin [37]. Mice in the sham, sham-VEH and sham-BBG groups received dural injections of PBS as vehicle. IS or PBS was injected into the previously implanted guide cannula using a microinjector (Hamilton) with an injection volume of 20μL, once a day for 4 days. The dose of IS was chosen based on our previous studies and was confirmed to induce a migraine-like phenotype in mice [34, 37, 38]. Extreme care should be taken to avoid irritation of the dura mater when using the microinjector. The IS or PBS in the guide tube will continuously stimulate the dura mater through the pre-drilled skull hole until the next dural injection.

#### Intraperitoneal injection

The P2X7R selective antagonist, BBG (Sigma-Aldrich), was used to investigate the role of P2X7R in the IS-induced migraine mouse model. Mice in the sham-BBG and IS-BBG groups received intraperitoneal injection of BBG. BBG solution was prepared freshly each day with 0.9% saline and intraperitoneal injection at 50 mg/kg/day, 30 min before dural injection, once a day for 4 days. The BBG dose was chosen based on previous studies [12, 39]. Mice in the sham-VEH and IS-VEH groups received intraperitoneal injection with an equivalent volume of 0.9% saline as vehicle. Detailed drug administration and experimental group were shown in Fig. 1.

### Sample collection and processing

The mice were euthanized by inhaling a lethal dose of carbon dioxide (CO_2_) and their brains were harvested. Their cerebral cortex and hippocampus were immediately separated on ice and were frozen in dry ice for immunoblot (*n* = 6 in each experimental group) and enzyme-linked immunosorbent assay (ELISA) (*n* = 4 in each experimental group). Another group of animals were sacrificed for histological analysis (*n* = 6 in each experimental group). Mice were deeply anaesthetized and transcardial perfusion with 40 mL of 0.9% chilled saline then followed by 40 mL of 4% chilled paraformaldehyde. After perfusion, the brains were harvested and fixed overnight in vials containing 4% paraformaldehyde solution.

### Immunoblot analysis

Brain tissues were homogenized in RIPA buffer (G2002, Servicebio) containing protease inhibitor cocktail (G2006, Servicebio) and phenylmethylsulfonyl fluoride (PMSF, G2008, Servicebio). Protein samples were separated using 8–12% sodium dodecyl sulfate–polyacrylamide gel electrophoresis (SDS-PAGE), and then transferred onto polyvinylidene-difluoride (PVDF) membrane to probe for proteins. After being blocked with 5% skimmed milk powder, the PVDF membranes were incubated with the following primary antibodies: P2X7 (1:500, APR-004, Alomone labs), NLRP3 (1:1000, AG-20B-0014, Adipogen), total caspase-1 and cleaved caspase-1 (1:1000, AG-20B-0042, Adipogen), total caspase-3 and cleaved caspase-3 (1:1000, 19,677–1-AP, Proteintech), full length GSDMD (1:1000, ab219800, abcam), N-terminal of GSDMD (1:1000, ab209845, abcam) and β-actin (1:5000, 20,536–1-AP, Proteintech) overnight at 4 °C with agitation. After primary antibody incubation, membranes were washed with 1xTBST (3 times for 10 min each) before incubating with horseradish peroxidase (HRP)-conjugated secondary antibodies (Goat Anti Rabbit, 1:5000, SA00001-2, Proteintech; Goat Anti Mouse, SA00001-1, 1:5000, Proteintech) for 1 h at room temperature with agitation. Then membranes were washed with 1xTBST (3 times for 10 min each). The substrate for HRP, enhanced chemiluminescence (BL523B, Biosharp) was applied before the membranes were visualized using chemiluminescence imaging system (BioRad Laboratories). The chemiluminescence intensity of protein bands was measured using Image J software (Version 1.46; National Institute of Health, Bethesda, MD, USA), and the relative expression of proteins was normalized to the corresponding β-actin.

### ELISA

Proinflammatory cytokines (IL-1β and IL-18) were quantified using ELISA. Brain tissues were homogenized on ice in phosphate buffer saline (PBS, pH 7.4) containing protease inhibitors. Tissue homogenates were centrifuged at 12,000 rpm for 15 min at 4℃. The supernatant was then centrifuged at 12,000 rpm for another 15 min at 4℃. The protein concentration of the supernatant of each sample was measured using bicinchonic acid (BCA) assay. ELISA kit for measuring IL-1β (catalog number: MLB00C; R&D Systems) and IL-18 (catalog number: ML002294, Mlbio) were used to quantify its content in the samples according to the manufacturer’s instructions. The quantity of IL-1β and IL-18 in each brain sample were standardized to the protein concentration.

### Immunofluorescence analysis

After post-fixed in 4% paraformaldehyde solution overnight at 4℃, mouse brain tissues were processed into paraffin wax blocks and then cut into coronal Sects. (5 µm-thick). Paraffinized brain sections were dewaxed, hydrated and heat-induced antigen retrieval. Then all brain sections were permeabilized with 0.3% Triton X-100 solution for 10 min and blocked with 5% bovine serum albumin (BSA) solution for 1 h. The sections were immunostained with P2X7 (1:50, APR-004, Alomone labs), caspase-1 (1:200, AG-20B-0042, Adipogen), caspase-3 (1:200, 19,677–1-AP, Proteintech), GSDMD (1:500, ab219800, abcam), microtubule-associated protein 2 (MAP2, 1:200, GB11128-2, Servicebio), myelin basic protein (MBP, 1:200, GB12226, Servicebio), neuronal nuclei (NeuN, 1:200, ab104224, abcam), glial fibrillary acidic protein (GFAP, 1:200, GTX40988, GeneTex), ionized calcium-binding adaptor molecule-1 (Iba-1, 1:500, 019–19,741, Wako) overnight at 4 °C. Subsequently, they were incubated with FITC- (Goat Anti Rabbit, 1:200, GB22303, Servicebio; Goat Anti Mouse, 1:200, GB22301, Servicebio) or CY3- (Goat Anti Rabbit, 1:200, GB21303, Servicebio; Goat Anti Mouse, 1:200, GB21301, Servicebio) conjugated secondary antibodies for 1 h at room temperature in complete darkness. Furthermore, the nuclei were stained using DAPI (G1012, Servicebio). Images were captured under × 20, × 40 and × 100 magnification using an Olympus upright Fluorescence Microscope BX53. Morphological and quantitative analysis were performed in a blinded manner using Image J software (Version 1.46; National Institute of Health, Bethesda, MD, USA). For quantification of positively staining, three randomly microscopic fields were acquired in each section and three adjacent sections were examined from each mouse. The average percentage of the positively stained area was calculated to reflect the degree of positive immunoreactivity.

### Cresyl violet staining and luxol fast blue

Cresyl violet staining was performed to detect the neuronal loss in the cerebral cortex and hippocampus. In brief, de-waxed rehydrated sections were immersed in Cresyl Violet solution (G1036, Servicebio) for 5 min and washed in ultrapure water. Then the sections were dehydrated in a graded series of ethanol (70–100%) and finally cleared in xylene. Luxol-fast-blue (LFB) staining was performed in order to evaluate the white matter integrity. Sections were immersed in the LFB solution (G1030, Servicebio) at 37℃ overnight. The excess staining was first removed with 95% ethanol and then washed by ultrapure water. Finally, to differentiate the white matter from the gray matter, the sections were immersed in 0.05% aqueous lithium carbonate for 20 s followed by 70% ethanol until the nuclei are decolorized. The bright field images were captured using an Olympus upright Fluorescence Microscope BX53 with a 4x, 20 × or 40 × objective lens. The number of Nissl-positive cells in cerebral cortex and hippocampal CA1, CA2, CA3, dentate gyrus (DG) regions was used to represent the severity of neuronal loss in the corresponding brain regions. The number of Nissl-positive cells was counted by three examiners who were blinded to experimental conditions, as previously described [27]. The white matter of five brain regions was evaluated: the corpus callosum (Medial), corpus callosum (Paramedian), caudoputamen, internal capsule and optic tract. The severity of the white matter lesions was classified into three levels: Grade 0-normal, Grade 1- disarrangement of the nerve fibers, Grade 2—the formation of visible vacuoles, Grade 3—the disappearance of myelinated fibers.

### Behavioral test

#### Von frey testing

In order to habituate to testing environment, mice were placed in testing chambers for 2 h/day for 3 consecutive days prior to the first test. Baseline mechanical pain thresholds were assessed before the first drug administration. Mice with a facial baseline threshold above 0.6 g (g) were included in the study. Subsequently, these animals were randomly assigned to different experimental groups and received corresponding drug administrations. Post-treatment mechanical pain thresholds were assessed 1 h after the last drug administration. Von Frey filament thresholds were determined by the Dixon “up-and-down” method [40]. Testing began with the 0.008 g filament on the periorbital region and gradually increased until the mice showed a positive reaction, which referred to quick withdrawal of its head in response to the stimulus. Each mouse was tested at least three times, and there was a 1–2 min interval between consecutive stimuli. All investigators were blinded to experimental conditions.

#### Head-scratching

Head-scratching was considered to be a manifestation of spontaneous pain behavior in mice, referring to unilateral or bilateral forepaw scratching of the area innervated by the V1 branch of the trigeminal nerve (including the scalp and periorbital area) [37]. Notably, some movements of mice do not indicate pain, such as head-shaking, grooming and yawning, etc. The number of head-scratching was counted during 1-h period immediately after the last drug administration. The work was conducted prior to post-treatment Von Frey testing to avoid the effects of filament stimulations on the behavior of mice. All investigators were blinded to experimental conditions.

#### Novel object recognition assay

Novel object recognition test was performed to evaluate recognition memory of mice. The test was carried out in an open field apparatus (60 cm long x 40 cm wide x 60 cm high). In the habituation phase (day 1), each mouse was left alone in the apparatus without any objects for 30 min to acclimatize to the environment. In the training phase (day 2), two identical objects were placed in opposite corners of the apparatus 10 cm from the walls. Each mouse was allowed to explore two identical objects for 10 min. In the test phase (day 3), one of the two objects was changed to a novel one, which was different from the former object in both color and shape. Each mouse was allowed to explore two different objects for 10 min. The mouse’s nose directing toward the object at less than 2 cm and exploring it (i.e., interacting with the object or sniffing) was regarded as exploration of an object. Time spent exploring novel object (T_novel_) and old object (T_old_) was recorded. A discrimination ratio (Discrimination Ratio = T_novel_ – T_old_ / T_novel_ + T_old_) was calculated to reflect the preference for exploring the novel object [41].

#### Morris water maze assay

Morris water maze was conducted to assess spatial learning and memory of mice. On day 1 (visible platform), mice were trained to find an above-water platform with a visible cue. Each mouse completed 4 trials a day. There is a 15-min interval between each trial. In each trial, the starting direction was different, but the location of the platform was fixed. The escape latency to reach the platform was recorded and the average of 4 trials was calculated. When the platform was found within 60 s, the mouse was allowed to stay on the platform for 10 s. If the platform was not found within 60 s, the mouse would be guided to the platform, allowed to stay on the platform for 15 s and the escape latency was recorded as 60 s. On day 2–5 (invisible platform), mice were trained to find an invisible, submerged platform following the same procedure as day 1. On day 6 as probe trial (no platform), the platform was removed and the mice were allowed to swim freely in the pool for 60 s. Time spent in target quadrant and the number of platform crossings were recorded. All trials were recorded and analyzed using the ANY-Maze software (San Diego Instruments).

### Statistical analysis

We used R software (R version 3.6.1) for statistical analysis. All values were expressed as mean ± standard error of the mean (SEM), and tested for normality using the Shapiro–Wilk test. Normally distributed datasets were analyzed using unpaired, two-tailed Student’s t tests for two groups (Experiment 1). Comparisons among the four groups were carried out using one-way or two-way analysis of variance (ANOVA) (Experiment 2). Post hoc multiple comparisons were conducted using Tukey’s test. Nonparametric distribution datasets were analyzed using Mann–Whitney U test for two groups and Kruskal–Wallis test for four groups followed by Dunn’s multiple comparisons test. For training phase of the Morris water maze, statistical analysis was performed using a repeated ANOVA. Statistical significance was set as a *p* value < 0.05.

## Results

### Repeated dural IS stimulation activated the P2X7R-NLRP3 signaling pathway

**Fig. 2:**
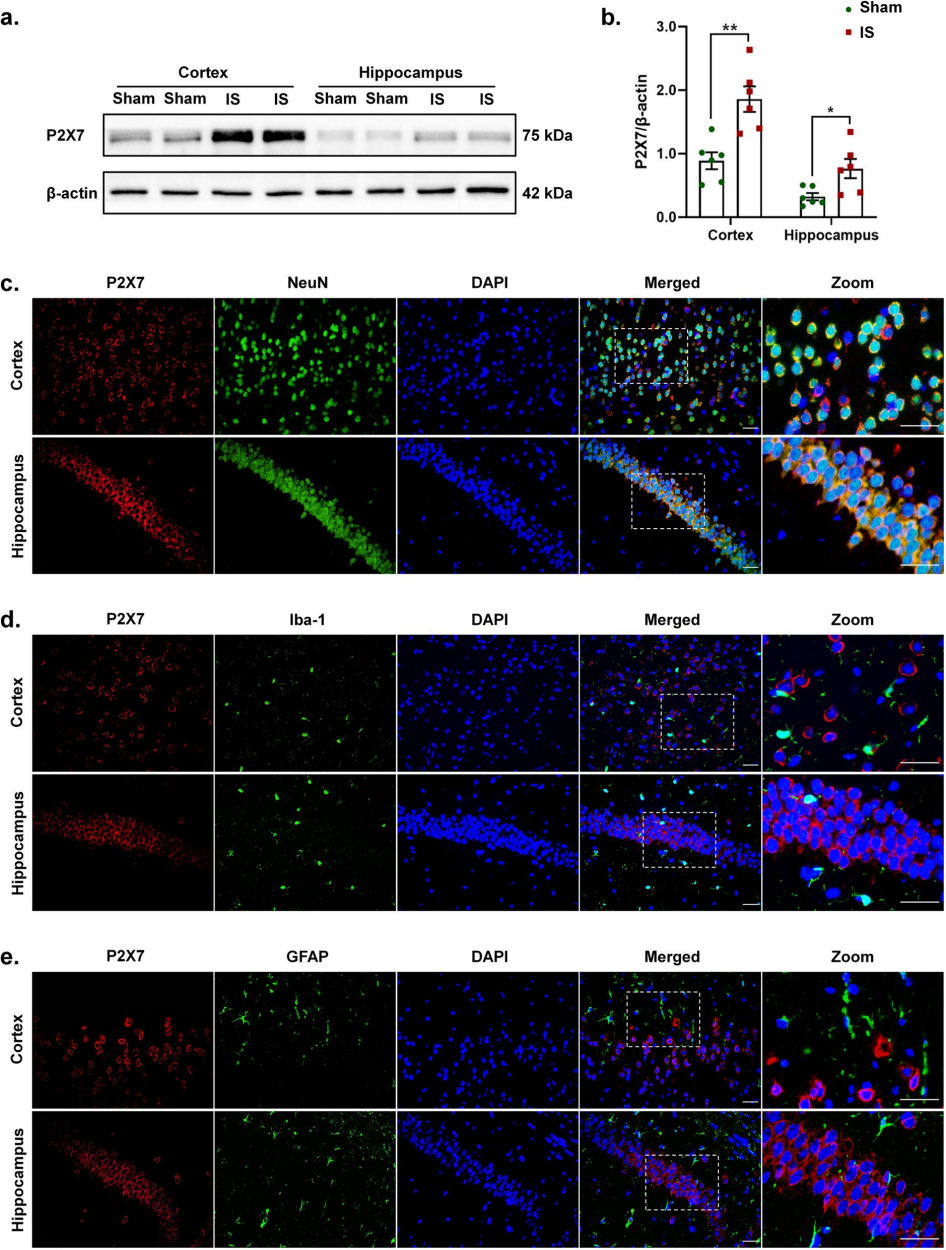
Repeated dural IS stimulation upregulated P2X7R in the cortical and hippocampal neurons. (**a**-**b**) Representative immunoblots and quantification illustrated increased levels of P2X7R in the cerebral cortex and hippocampus after repeated dural IS stimulation. (**c**-**e**) Double immunofluorescence staining showed that P2X7R was co-localized within neurons (NeuN positive) in the cerebral cortex and hippocampus. No substantial colocalization of P2X7R was observed in microglia (Iba-1 positive) and astrocytes (GFAP positive) in the cortex and hippocampus. Magnification × 40. Insets show a higher magnification view (Zoom). Scale bar, 20 μm. Images were taken under identical exposures and conditions. *n* = 6 mice per group. ^*^p < 0.05, ^**^p < 0.01 and ^***^p < 0.001 as compared to sham mice. Datasets in (b) were analyzed using Student’s t test. Data are represented as mean ± SEM. Abbreviations: IS, inflammatory soup; NeuN, neuronal nuclei; Iba-1, ionized calcium-binding adaptor molecule-1; GFAP, glial fibrillary acidic protein

**Fig. 3:**
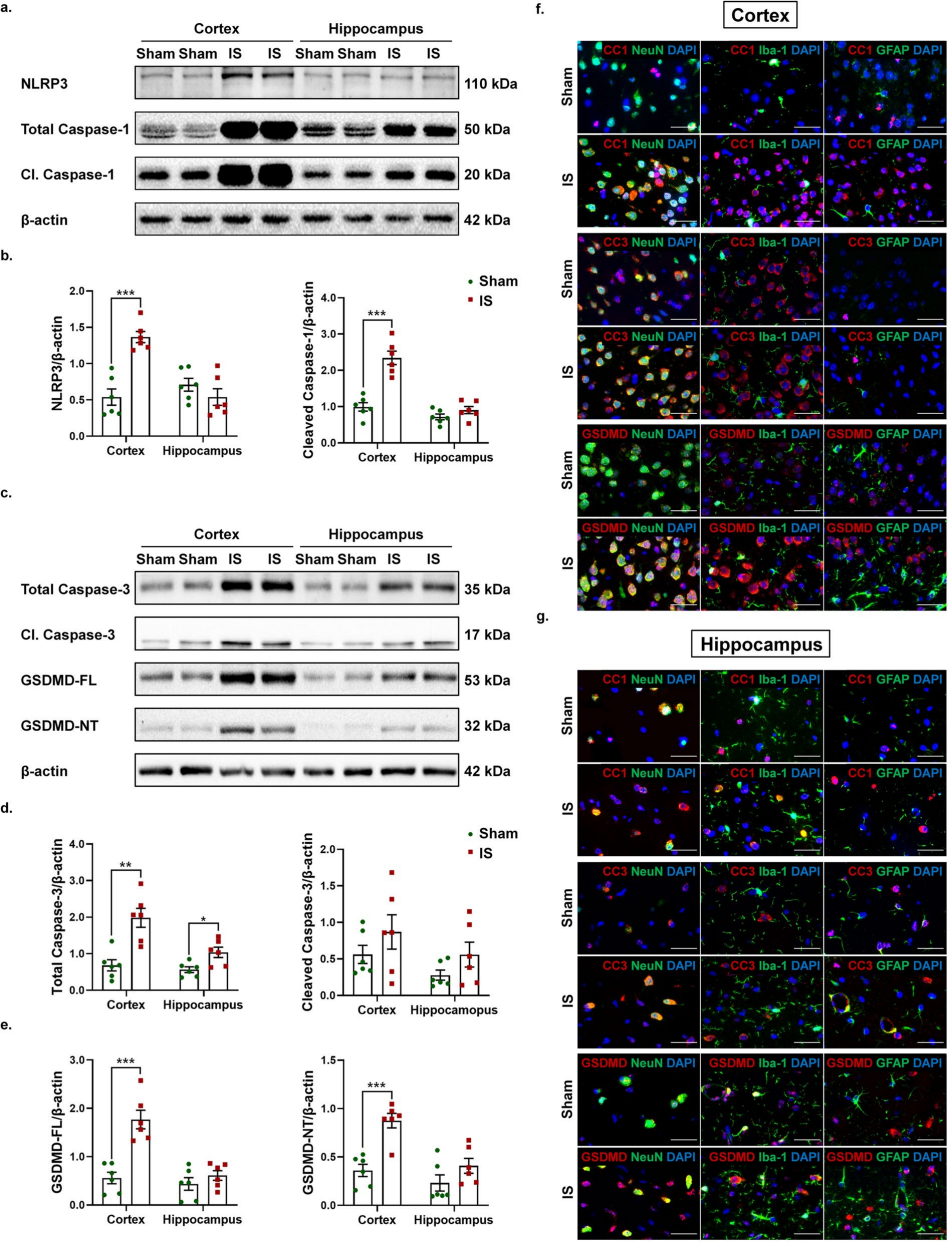
Repeated dural IS stimulation activated NLRP3 inflammasome and pyroptosis in the cortical and hippocampal neurons and microglia. (a-b) Representative immunoblots and quantification illustrated increased expression levels of NLRP3 (inflammasome receptor) and cleaved caspase-1 (marker of inflammasome activation) in the cerebral cortex after repeated dural IS stimulation. (**c-e**) Representative immunoblots and quantification illustrated increased expression levels of GSDMD-NT (pyroptotic marker) in the cerebral cortex after repeated dural IS stimulation. The difference in cleaved caspase-3 (apoptotic marker) did not reach a statistically significant level. (**f-g**) Double immunofluorescence staining showed that cleaved caspase-1 (CC1) and GSDMD were co-localized within the cortical and hippocampal neurons (NeuN positive) and microglia (Iba-1 positive), while cleaved caspase-3 (CC3) was co-localized within neurons (NeuN positive). Zoom magnification × 100. Scale bar, 20 μm. Images were taken under identical exposures and conditions. *n* = 6 mice per group. ^*^p < 0.05, ^**^p < 0.01 and ^***^p < 0.001 as compared to sham mice. Student’s t test was performed for statistical analysis. Data are represented as mean ± SEM. Abbreviations: IS, inflammatory soup; Cl. caspase-1, cleaved caspase-1; Cl. caspase-3, cleaved caspase-3; GSDMD-FL, full length Gasdermin D; GSDMD-NT, N-terminal Gasdermin D; CC1, cleaved caspase-1; CC3, cleaved caspase-3; NeuN, neuronal nuclei; Iba-1, ionized calcium-binding adaptor molecule-1; GFAP, glial fibrillary acidic protein

We detected the expression levels of the key proteins of P2X7R-NLRP3 signaling pathway in the cerebral cortex and hippocampus following repeated dural IS stimulation (Figs. 2 and 3). Expression of P2X7R (*p* < 0.01), NLRP3 (inflammasome receptor, *p* < 0.001), total caspase-1 (inflammasome priming, *p* < 0.01), cleaved caspase-1 (CC1, marker of inflammasome activation, *p* < 0.001) were all increased in the cerebral cortex, while only expression of P2X7R (*p* = 0.033) was increased in the hippocampus after dural IS stimulation (Figs. 2a-b and 3a-b). Proinflammatory cytokines IL-1β (*p* < 0.001) and IL-18 (*p* < 0.001), direct downstream products of inflammasome activation, were also higher in the cerebral cortex in IS mice than sham controls (Fig. 5e-f). These results indicated the activation of P2X7R-NLRP3 signaling pathway following repeated dural IS stimulation.

We further examined the cellular specificity of P2X7R and NLRP3 inflammasome activation using double immunofluorescence labeling. The P2X7R was mainly expressed in the membrane of NeuN-positive neurons in the cerebral cortex and hippocampus (Fig. 2c-e), while CC1 was mainly expressed in NeuN-positive neurons and Iba-1 positive microglia of cortex and hippocampus (Fig. 3f-g, Supplementary Figs. 1a, d and 2). These results indicated that the dural IS stimulation activated P2X7R on neurons, as well as inflammasome on microglia and neurons.

### Repeated dural IS stimulation promoted pyroptosis

The active CC1 can promote cell death by activating apoptosis and pyroptosis pathways [24, 25]. We next detected protein expression levels of cleaved caspase-3 (CC3, apoptotic marker) and N-terminal GSDMD (GSDMD-NT, pyroptotic marker) and their precursor proteins (Fig. 3c-e). GSDMD-NT expression was increased in the cerebral cortex after repeated dural IS stimulation (*p* < 0.001, Fig. 3c, e), but no significant changes in CC3 expression (Fig. 3c-d), suggesting that dural IS stimulation induced pyroptosis-predominant cell death in the cerebral cortex. Consistent with the cellular localization of CC1, GSDMD was also localized in NeuN-positive neurons and Iba-1-positive microglia of cortex and hippocampus (Fig. 3f-g, Supplementary Figs. 1c, f and 4), supporting the occurrence of inflammasome-mediating pyroptosis in cortical and hippocampal neurons and microglia of the IS-induced migraine mouse model.

### Repeated dural IS stimulation induced gliosis and neuronal loss in the cortex and hippocampus, but no obvious white matter damage

**Fig. 4:**
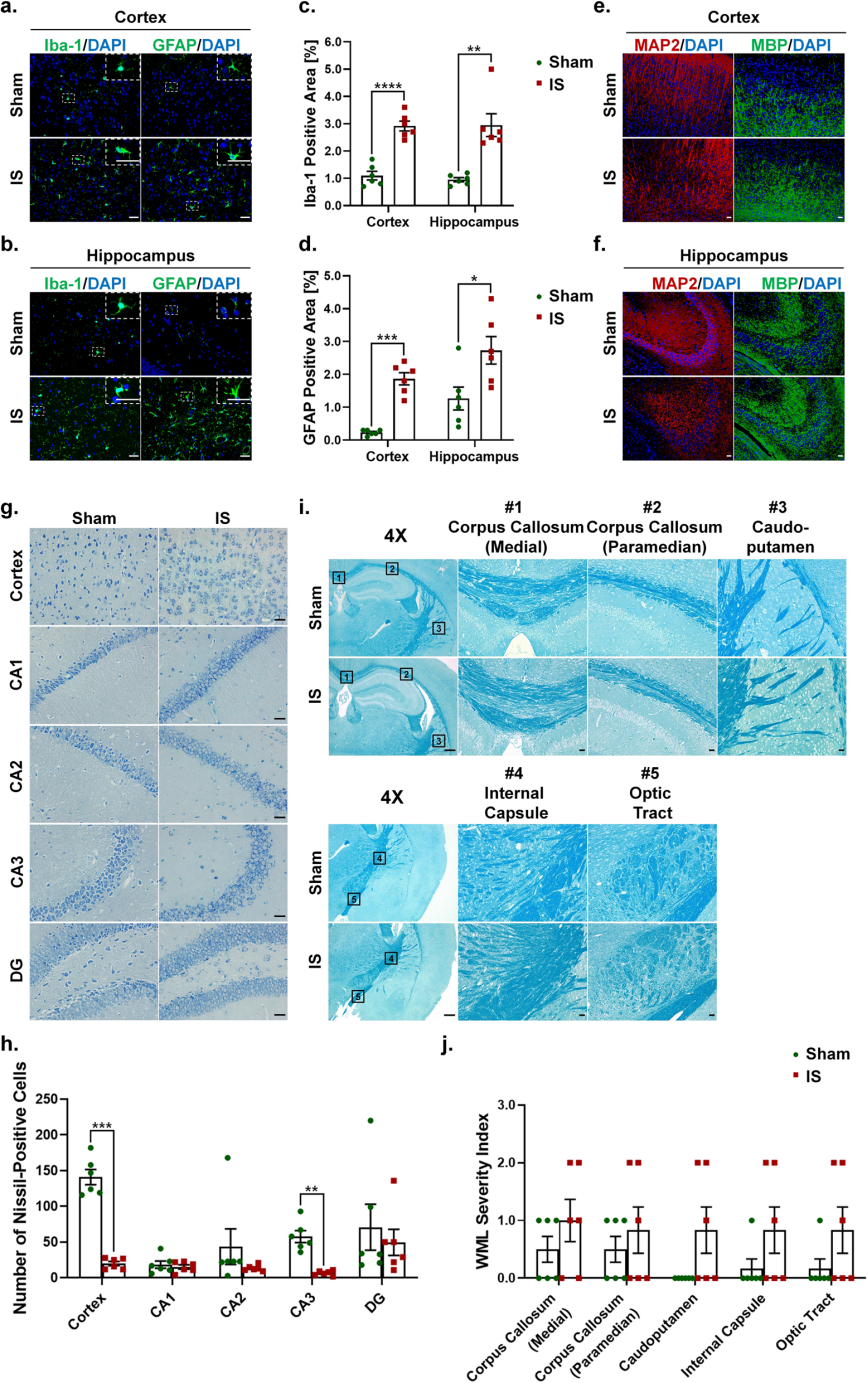
Repeated dural IS stimulation induced gliosis and neuronal loss in the cerebral cortex and hippocampus, but no obvious white matter damage. (**a**-**d**) Representative immunofluorescence analysis showed an increase in Iba-1 positive microglia and GFAP positive astrocytes in the cortex and hippocampus following dural IS stimulation, indicating microgliosis and astrogliosis. Magnification × 40. Insets show a higher magnification view. Scale bar, 20 μm. (**e**–**f**) Representative immunofluorescence images showed a decrease in MAP2-positive areas (an indicator of neuronal loss) in the cerebral cortex and hippocampus, but no significant change in MBP-positive areas (an indicator of white matter integrity) after repeated dural IS stimulation. Magnification × 20. Scale bar, 20 μm. (**g**-**h**) Representative crystal violet images and quantification showed the loss of Nissl-positive neurons in the cortical and hippocampal CA3 regions after repeated dural IS stimulation. Magnification × 40. Scale bar, 20 μm. (**i**-**j**) Representative Luxol fast blue stained images and quantification showed no obvious white matter lesion in the corpus callosum (medial), corpus callosum (paramedian), caudoputamen, internal capsule, and optic tract after repeated dural IS stimulation. The severity of white-matter disruption was graded accordingly: Grade 0 = no disruption; Grade 1 = disarrangement of nerve fibers; Grade 2 = formation of marked vacuoles; Grade 3 = disappearance of myelinated fibers. Magnification × 20. Scale bar, 20 μm. All images were taken under identical exposures and conditions. *n* = 6 mice per group. ^*^p < 0.05, ^**^p < 0.01 and ^***^p < 0.001 as compared to sham mice. Datasets in (j) were conducted with Mann–Whitney U test; the other datasets were analyzed using Student’s t test. Data are represented as mean ± SEM. Abbreviations: IS, inflammatory soup; Iba-1, ionized calcium-binding adaptor molecule-1; GFAP, glial fibrillary acidic protein; MAP2, microtubule-associated protein 2; MBP, myelin basic protein

To elucidate the mechanisms by which the P2X7R-NLRP3 signaling pathway mediates IS-induced cognitive impairment, we examined cognition-related pathological changes including gliosis (microglia and astrocyte) and neuronal loss in the cerebral cortex and hippocampus, and white matter damage. Mice receiving dural IS stimulation exhibited microgliosis and astrogliosis in the cerebral cortex and hippocampus, indicated by increased immunoreactivity of Iba-1 (marker of microglia) and GFAP (marker of astrocyte) (Fig. 4a-d). The cerebral cortex and hippocampus of IS mice also underwent neuronal loss, evidenced by reduced immunoreactivity of MAP2 and decreased Nissl-positive cells in cresyl violet staining (Fig. 4e-f, g-h). The white matter damage was assessed by MBP and LFB staining. No obvious white matter lesions were observed in the cerebral cortex and hippocampus measured by MBP staining and five white matter regions (including medial corpus callosum, paramedian corpus callosum, caudoputamen, internal capsule and optic tract) measured by LFB staining after repeated dural IS stimulation (Fig. 4e-f, i-j). These findings indicated that repeated dural IS stimulation induces microgliosis, astrogliosis and neuronal loss in the cerebral cortex and hippocampus, but no significant white matter damage.

### Inhibition of P2X7R attenuated IS-induced NLRP3 inflammasome activation and pyroptosis

**Fig. 5:**
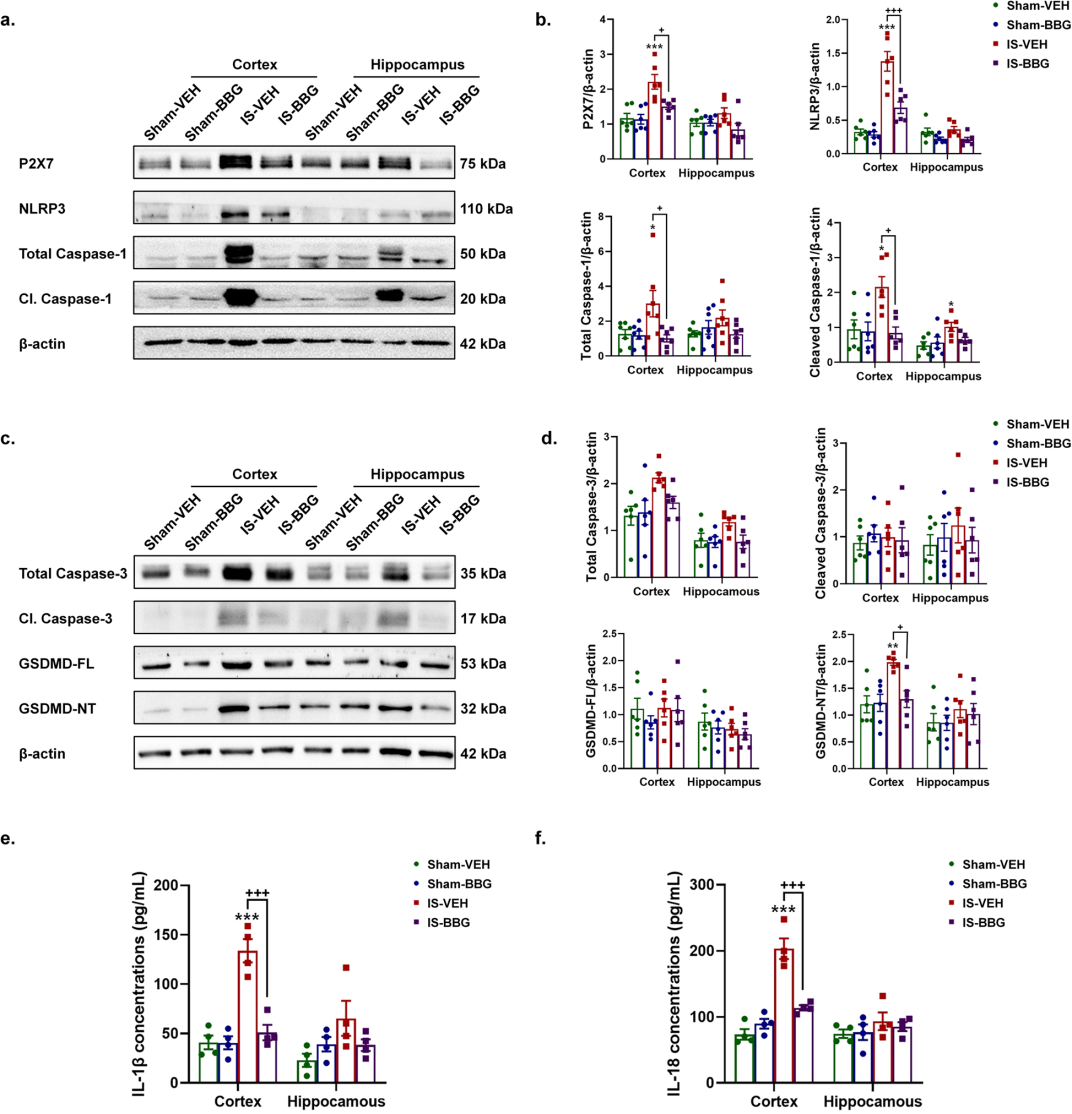
Inhibition of P2X7R attenuated IS-induced NLRP3 inflammasome activation and pyroptosis. (**a**-**b**) Representative immunoblots and quantification showed decreased expression levels of P2X7R, NLRP3 (inflammasome receptor) and cleaved caspase-1 (marker of inflammasome activation) in the cerebral cortex of IS-BBG mice compared to IS-VEH mice (*n* = 6 mice per group). (**c**-**d**) Representative immunoblots and quantification showed decreased expression levels of GSDMD-NT (pyroptotic marker) in the cerebral cortex of IS-BBG mice compared to IS-VEH mice (*n* = 6 mice per group). (**e**–**f**) ELISA results showed decreased release of IL-1β and IL-18 (direct downstream products of inflammasome activation) in the cerebral cortex of IS-BBG mice compared to IS-VEH mice (*n* = 4 mice per group). ^*^p < 0.05, ^**^p < 0.01 and ^***^p < 0.001 as compared to sham-VEH mice; ^+^*p* < 0.05, ^++^*p* < 0.01 and ^+++^*p* < 0.001 as compared to IS-VEH mice. One-way ANOVA with Tukey’s multiple comparisons or Kruskal–Wallis with Dunn’s multiple comparisons test were performed for statistical analysis. Data are represented as mean ± SEM. Abbreviations: IS, inflammatory soup; BBG, brilliant blue G; VEH, vehicle; GSDMD-NT, N-terminal Gasdermin D; ELISA, enzyme-linked immunosorbent assay

**Fig. 6:**
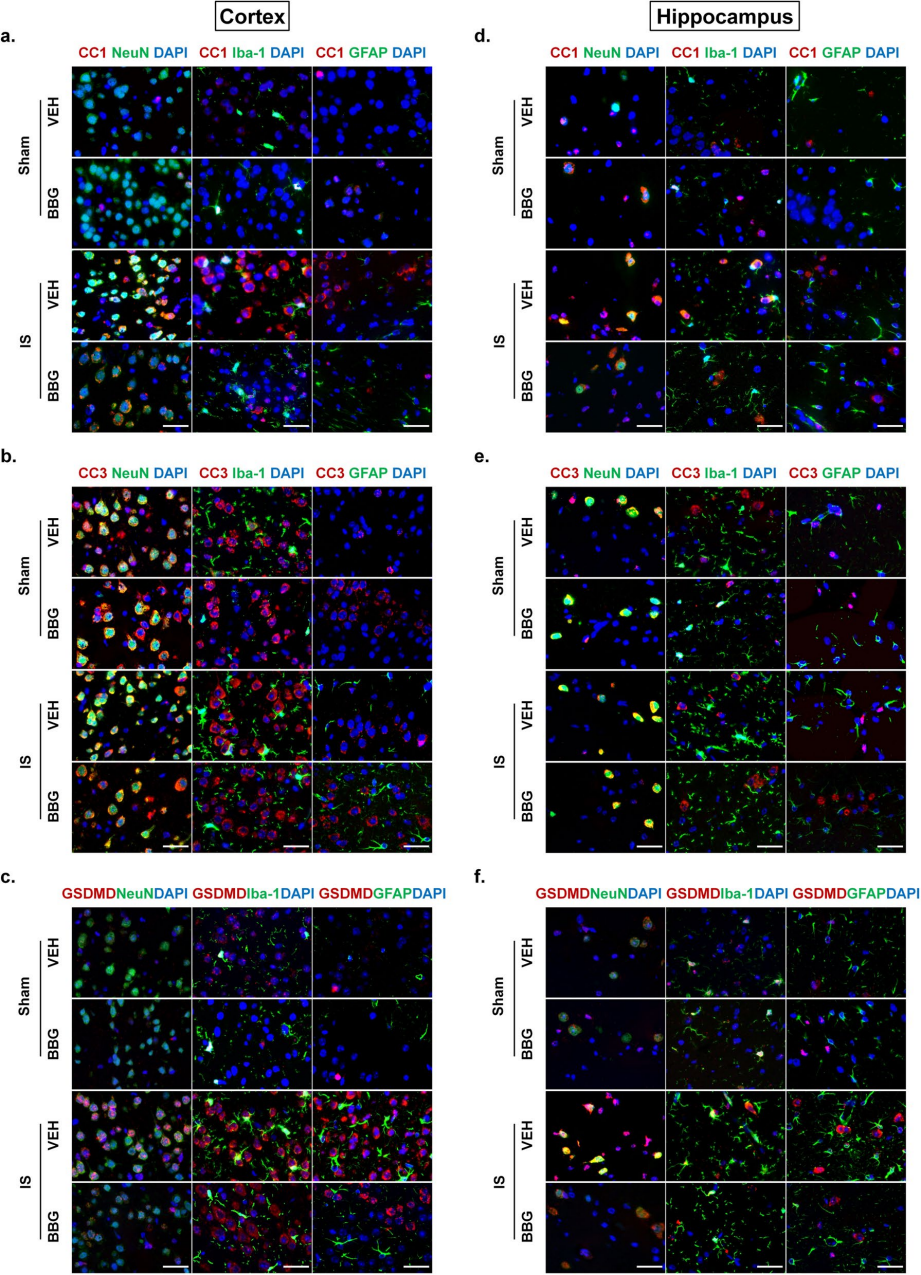
Inhibition of P2X7R attenuated IS-induced NLRP3 inflammasome activation and pyroptosis on neurons and microglia in the cerebral cortex and hippocampus. (**a, d**) Double immunofluorescence staining showed a reduction in CC1-positive (marker of inflammasome activation) neurons (NeuN positive) and microglia (Iba-1 positive) in the cerebral cortex and hippocampus of IS-BBG mice compared to IS-VEH mice. (**b, e**) Double immunofluorescence showed similar CC3-positive (apoptotic marker) neurons (NeuN positive) in the cerebral cortex and hippocampus of the four groups of mice. (**c, f**) Double immunofluorescence staining showed a reduction in GSDMD-positive (pyroptotic marker) neurons (NeuN positive) and microglia (Iba-1 positive) in the cerebral cortex and hippocampus of IS-BBG mice compared to IS-VEH mice. Magnification × 100. Scale bar, 20 μm. Images were taken under identical exposures and conditions. Abbreviations: IS, inflammatory soup; CC1, cleaved caspase-1; CC3, cleaved caspase-3; GSDMD, Gasdermin D; NeuN, neuronal nuclei; Iba-1, ionized calcium-binding adaptor molecule-1; GFAP, glial fibrillary acidic protein; BBG, brilliant blue G; VEH, vehicle

Given the upregulation of P2X7R and activation of its downstream NLRP3 inflammasome signaling pathway in the IS-induced migraine mice model, inhibition of P2X7R with BBG (a specific P2X7R antagonist) is a potential effective therapeutic approach. The IS-induced elevation of P2X7R expression was conspicuously restrained with the administration of BBG (Fig. 5a-b), suggesting that the P2X7R was effectively inhibited. The IS-induced NLRP3 inflammasome activation was attenuated after inhibition of P2X7R, evidenced by a decrease in protein expression levels of the components of NLRP3 inflammasome (NLRP3, total caspase-1) and downstream products of its activation (CC1, IL-1β and IL-18) after application of BBG (Fig. 5a-b, e–f). These findings supported the hypothesis that the IS-induced NLRP3 inflammasome activation was mediated by P2X7R. Furthermore, the expression of pro-pyroptotic protein (GSDMD-NT) was lower of IS-BBG mice than IS-VEH mice (Fig. 5c-d), indicating the IS-induced pyroptosis was attenuated after inhibition of P2X7R. The decreased number of CC1-positive and GSDMD-positive neurons and microglia in the IS-BBG mice compared to IS-VEH mice provided evidence for the role of P2X7R in the inflammasome activation and pyroptosis in neurons and microglia (Fig. 6a, c, d, f, Supplementary Figs. 5a, c, d, f, 6 and 8).

### Inhibition of P2X7R was resistant to IS-induced gliosis and neuronal loss

**Fig. 7:**
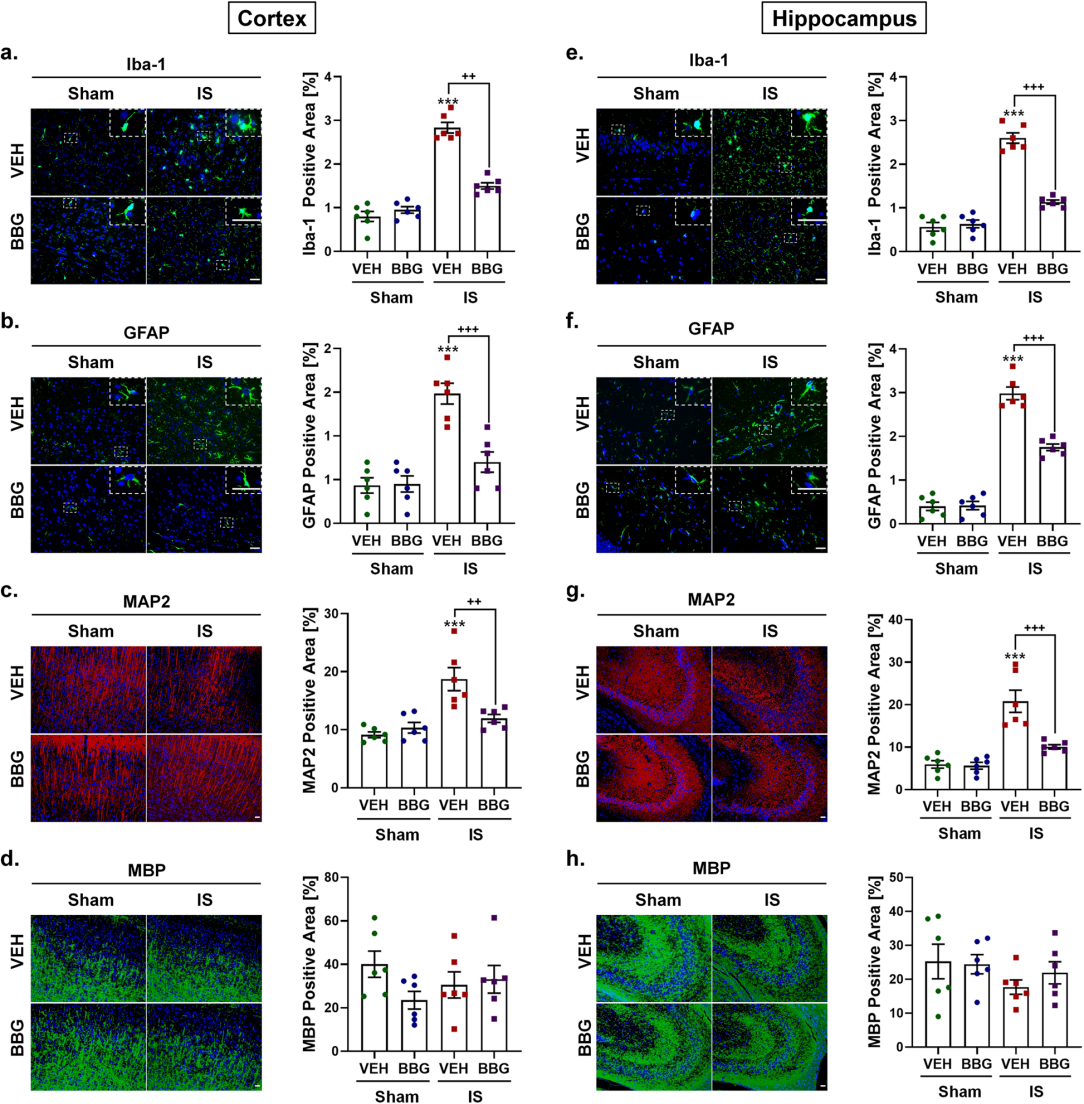
Inhibition of P2X7R attenuated IS-induced gliosis and neuronal loss in the cerebral cortex and hippocampus. (**a, b, e, f**) Representative immunofluorescence and quantification of Iba-1 and GFAP illustrating resistance to microgliosis and astrogliosis due to decreased expression of Iba-1and GFAP in the cerebral cortex and hippocampus of IS-BBG mice compared to IS-VEH mice. Magnification × 40. Insets show a higher magnification view. Scale bar, 20 μm. (**c, g**) Representative immunofluorescence and quantification of MAP2 illustrating resistance to neuronal loss due to decreased expression of MAP2 in the cerebral cortex and hippocampus of IS-BBG mice compared to IS-VEH mice. Magnification × 20. Scale bar, 20 μm. (**d, h**) Representative immunofluorescence and quantification of MBP illustrating no significant difference in white-matter integrity in the cerebral cortex and hippocampus among the four groups. Magnification × 20. Scale bar, 20 μm. All images were taken under identical exposures and conditions. *n* = 6 mice per group. ^*^p < 0.05, ^**^p < 0.01 and ^***^p < 0.001 as compared to sham-VEH mice; ^+^*p* < 0.05, ^++^*p* < 0.01 and ^+++^*p* < 0.001 as compared to IS-VEH mice. One-way ANOVA with Tukey’s multiple comparisons or Kruskal–Wallis with Dunn’s multiple comparisons test were performed for statistical analysis. Data are represented as mean ± SEM. Abbreviations: IS, inflammatory soup; BBG, brilliant blue G; VEH, vehicle; Iba-1, ionized calcium-binding adaptor molecule-1; GFAP, glial fibrillary acidic protein; MAP2, microtubule-associated protein 2; MBP, myelin basic protein

**Fig. 8:**
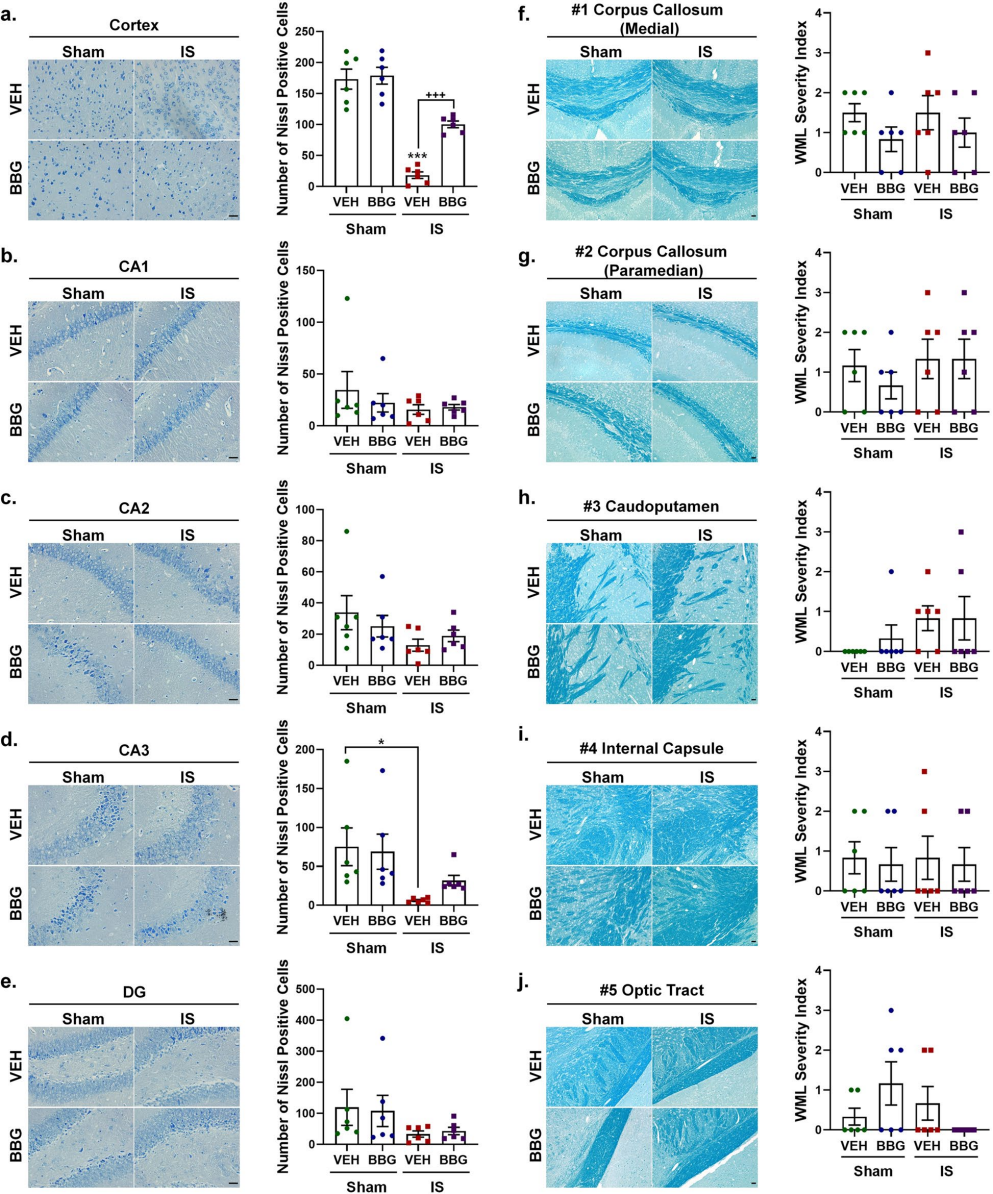
Inhibition of P2X7R attenuated IS-induced neuronal loss in the cerebral cortex and hippocampus. (**a**-**e**) Representative crystal violet images and quantification illustrating increased Nissl-positive neurons in the cerebral cortex of IS-BBG mice compared to IS-VEH mice. The number of Nissl-positive neurons in hippocampal CA1, CA2, CA3 and DG was similar between IS-BBG mice and IS-VEH mice. Magnification × 40. Scale bar, 20 μm. (**f**-**j**) Representative Luxol fast blue stained images and quantification illustrating no significant difference in white-matter integrity in the corpus callosum (medial), corpus callosum (paramedian), caudoputamen, internal capsule and optic tract among the four groups. Magnification × 20. Scale bar, 20 μm. All images were taken under identical exposures and conditions. *n* = 6 mice per group. ^*^p < 0.05, ^**^p < 0.01 and ^***^p < 0.001 as compared to sham-VEH mice; ^+^*p* < 0.05, ^++^*p* < 0.01 and ^+++^*p* < 0.001 as compared to IS-VEH mice. One-way ANOVA with Tukey’s multiple comparisons or Kruskal–Wallis with Dunn’s multiple comparisons test were performed for statistical analysis. Data are represented as mean ± SEM. Abbreviations: IS, inflammatory soup; BBG, brilliant blue G; VEH, vehicle; DG, dentate gyrus

We further explored the role of P2X7R in IS-induced cognition-related pathological changes (i.e., microgliosis, astrogliosis and neuronal loss). We found that inhibition of P2X7R was resistant to IS-induced gliosis, indicated by the lower immunoreactivity of Iba-1 (marker of microglia) and GFAP (marker of astrocyte) in the cortex and hippocampus of the IS-BBG mice than IS-VEH mice (Fig. 7a-b, e–f). Inhibition of P2X7R was also resistant to IS-induced by neuronal loss, evidenced by the IS-induced loss of MAP2- and Nissl-positive cells was partially reversed after administration of BBG (Figs. 7c, g and 8a, d). The role of P2X7R in white matter integrity was assessed by MBP and LFB staining. Immunoreactivity of MBP and the white matter lesions index measured by LFB staining displayed no difference among the four groups, including sham-VEH, sham-BBG, IS-VEH and IS-BBG group (Figs. 7d, h and 8f-j). These results indicated that the IS-induced gliosis and neuronal loss were associated with the IS-induced upregulation of P2X7R.

### Inhibition of P2X7R prevented IS-induced nociceptive behavior and cognitive deficits

**Fig. 9:**
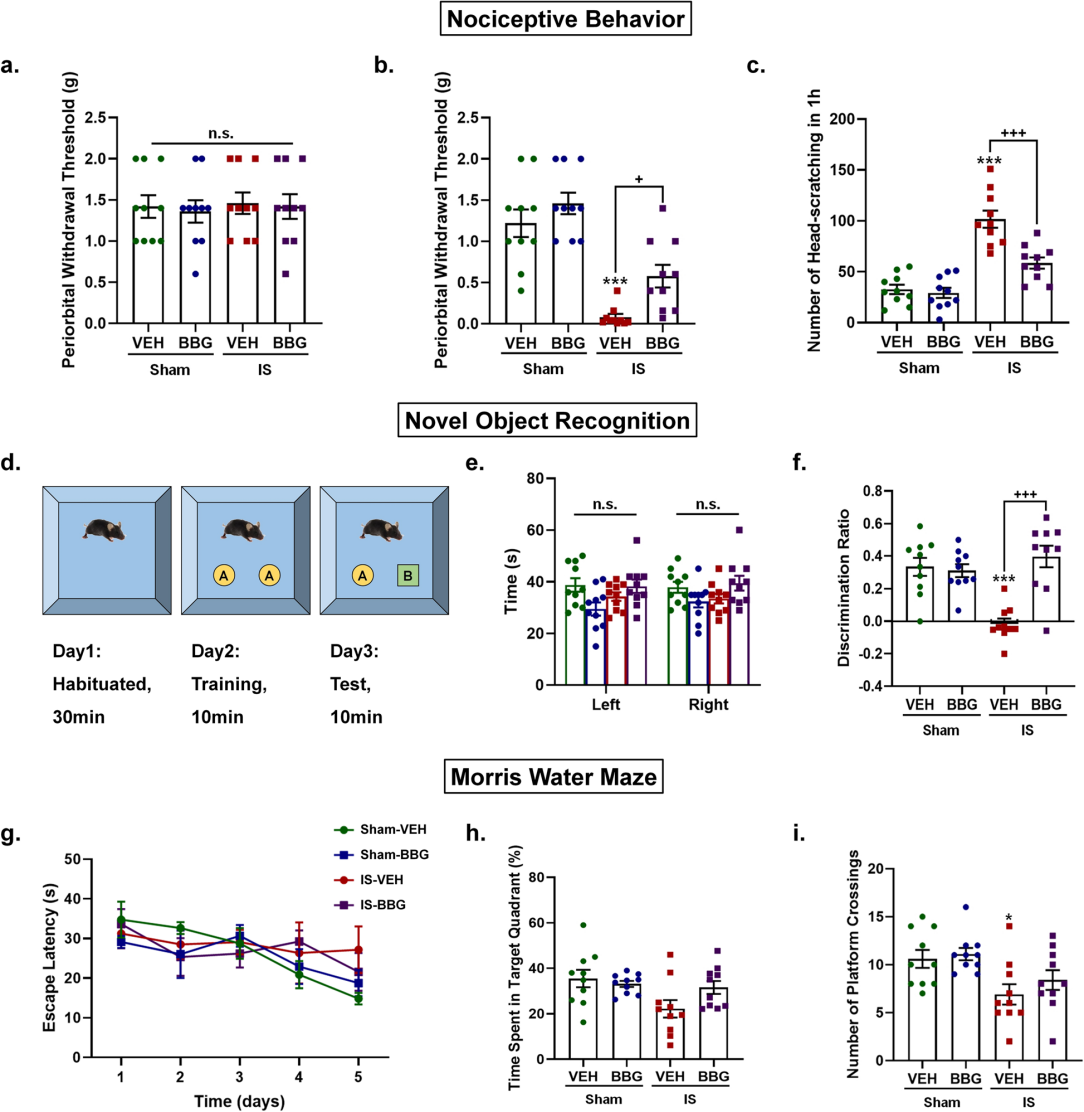
Inhibition of P2X7R prevented IS-induced nociceptive behavior and cognitive deficits. **a**-**c** Nociceptive behaviors were assessed using periorbital withdrawal threshold and the number of head-scratching within 1 h. (**a**) Baseline periorbital withdrawal thresholds measured before the first drug administration were not significantly different among the four groups. (**b**-**c**) The sham-VEH, sham-BBG and IS-BBG mice had higher post-treatment periorbital withdrawal thresholds measured 1-h after the last drug administration (**b**) and less head-scratching within 1-h measured immediately after the last drug administration (**c**) than IS-VEH mice, indicating the IS-induced nociceptive behaviors were partially prevented by pretreatment with BBG. **d**-**f** Non-spatial recognition memory was assessed using novel object recognition test. (**d**) Schematic illustration of the novel object recognition test. (**e**) All mice showed similar explorations of two identical objects at day 2 (training stage). (**f**) The sham-VEH, sham-BBG and IS-BBG mice showed a higher discrimination ratio than IS-VEH mice, indicating the IS-induced impairment of non-spatial recognition memory was improved by pretreatment with BBG. (Discrimination Ratio = T_novel_ – T_old_ / T_novel_ + T_old_). **g-i** Spatial learning and memory was assessed using Morris water maze test. Average latency to find visible platform on day 1 and hidden platform in target quadrant on day 2–5. All mice exhibited similar average latency to find a visible platform on day 1 (**g**), suggesting the similar vision and motor ability of all mice. From day 2 to day 5 (learning period), the escape latency gradually decreased in all mice and the slope of the decrease was not significantly different among the four groups (**g**), suggesting the similar spatial learning ability of all mice. On day 6 (probe trial), IS-VEH mice displayed a shorter time spent in target quadrant (**h**) and less platform crossings (**i**) than sham-VEH mice, suggesting the impaired spatial memory retention in IS-VEH mice. Although IS-BBG mice displayed a trend with a longer time spent in target quadrant (**h**) and more platform crossings (**i**) than IS-VEH mice, the difference did not reach statistical levels. *n* = 10 mice per group. ^*^p < 0.05, ^**^p < 0.01 and ^***^p < 0.001 as compared to sham-VEH mice; ^+^*p* < 0.05, ^++^*p* < 0.01 and ^+++^*p* < 0.001 as compared to IS-VEH mice. Repeated-measures ANOVA with Tukey’s multiple comparisons was performed for datasets in (g); the other datasets were analyzed using one-way ANOVA with Tukey’s multiple comparisons or Kruskal–Wallis with Dunn’s multiple comparisons test. Data are represented as mean ± SEM. Abbreviations: IS, inflammatory soup; BBG, brilliant blue G; VEH, vehicle; T_novel_, time spent in exploring the novel object; T_old_, time spent in exploring the old object

Finally, we examined the role of P2X7R in IS-induced nociceptive behavior and cognitive deficits. Nociceptive behavior was assessed by periorbital withdrawal thresholds and the number of head-scratching. There was no significant difference in baseline periorbital withdrawal thresholds measured before the first drug administration among four groups (Fig. 9a). The IS-VEH mice showed a significant decrease in post-treatment periorbital withdrawal thresholds measured 1-h after the last drug administration (*p* < 0.001, Fig. 9b) and an increase in head-scratching within 1-h measured immediately after the last drug administration (*p* < 0.001, Fig. 9c) compared to sham-VEH mice, indicating successful modeling of the IS-induced migraine mouse model. These IS-induced nociceptive behaviors were partially prevented by pretreatment with BBG, indicated by the higher post-treatment periorbital withdrawal thresholds (*p* = 0.04, Fig. 9b) and less head-scratching (*p* < 0.001, Fig. 9c) of IS-BBG mice than IS-VEH mice.

The cognitive functions of mice were assessed by novel object recognition assay (non-spatial recognition memory) and Morris water maze test (spatial learning and memory). In the novel object recognition assay, all mice showed similar explorations of two identical objects in the training stage (Fig. 9e). In the test stage, IS-VEH mice spent less time sniffing the novel object than sham-VEH mice (*p* < 0.001, Fig. 9f), suggesting a poor non-spatial recognition memory of IS-VEH mice. The impaired recognition memory of IS-VEH mice was improved after BBG application (IS-VEH *vs* IS-BBG: *p* < 0.001, Fig. 9f). As for the Morris Water Maze test, there was no difference in the average latency to find a visible platform on day 1 among the four groups of mice (Fig. 9g), suggesting the similar vision and motor ability of all mice. From day 2 to day 5 (learning period), the escape latency gradually decreased in all mice and the slope of the decrease was not significantly different among the four groups of mice (Fig. 9g), suggesting the similar spatial learning ability of all mice. On day 6 (probe trial), IS-VEH mice displayed a shorter time spent in target quadrant (*p* = 0.024, Fig. 9h) and less platform crossings (*p* = 0.038, Fig. 9i) than sham-VEH mice, suggesting the impaired spatial memory retention in IS-VEH mice. Although IS-BBG mice displayed a trend with a longer time spent in target quadrant (Fig. 9h) and more platform crossings (Fig. 9i) than IS-VEH mice, the difference did not reach statistical levels. These data showed that IS-induced cognitive impairment was characterized by impaired non-spatial recognition memory and spatial memory retention. Moreover, the impaired recognition memory can be improved by inhibition of P2X7R.

## Discussion

In this study, we used an IS-induced migraine mouse model to explore its effect on the P2X7R-NLRP3 signaling pathway and cognition. Our findings indicated that repeated dural IS stimulation triggered upregulation of P2X7R, activation of NLRP3 inflammasome, release of proinflammatory cytokines (IL-1β and IL-18) and activation of pyroptotic cell death pathway. Gliosis (microgliosis and astrogliosis), neuronal loss and cognitive impairment also occurred in the IS-induced migraine model. No significant apoptosis or whiter matter damage was observed following IS-induced migraine attacks. These pathological changes occurred mainly in the cerebral cortex and to a less extent in the hippocampus, all of which can be prevented by pretreatment with a specific P2X7R antagonist BBG. Moreover, BBG alleviated IS-induced cognitive impairment. These observations identified P2X7R as a key contributor to migraine-related cognitive impairment.

### Activation of P2X7R-NLRP3 signaling pathway following dural IS stimulation

P2X7R, a purinergic receptor family member, has been shown to be upregulated in TNC (a central area related to migraine pain) in a mouse model of NTG-induced migraine [12, 13]. Inhibition of P2X7R can alleviate NTG-induced mechanical and thermal hyperalgesia by negatively modulating the autophagic pathway and reducing proinflammatory cytokine IL-1β release [12, 13]. However, the role of P2X7R in migraine-related cognitive impairment has not been disclosed. Migraineurs frequently exhibited structural and functional abnormalities in the cerebral cortex and hippocampus, which is one of the possible mechanisms accounting for cognitive decline in migraineurs [5, 31]. In the present study, we observed the elevated expression level of P2X7R in cerebral cortex and hippocampus, brain regions related to cognition, following repeated dural IS stimulation (Fig. 2), suggesting a potential role of P2X7R in migraine-related cognitive deficit.

**Fig. 10:**
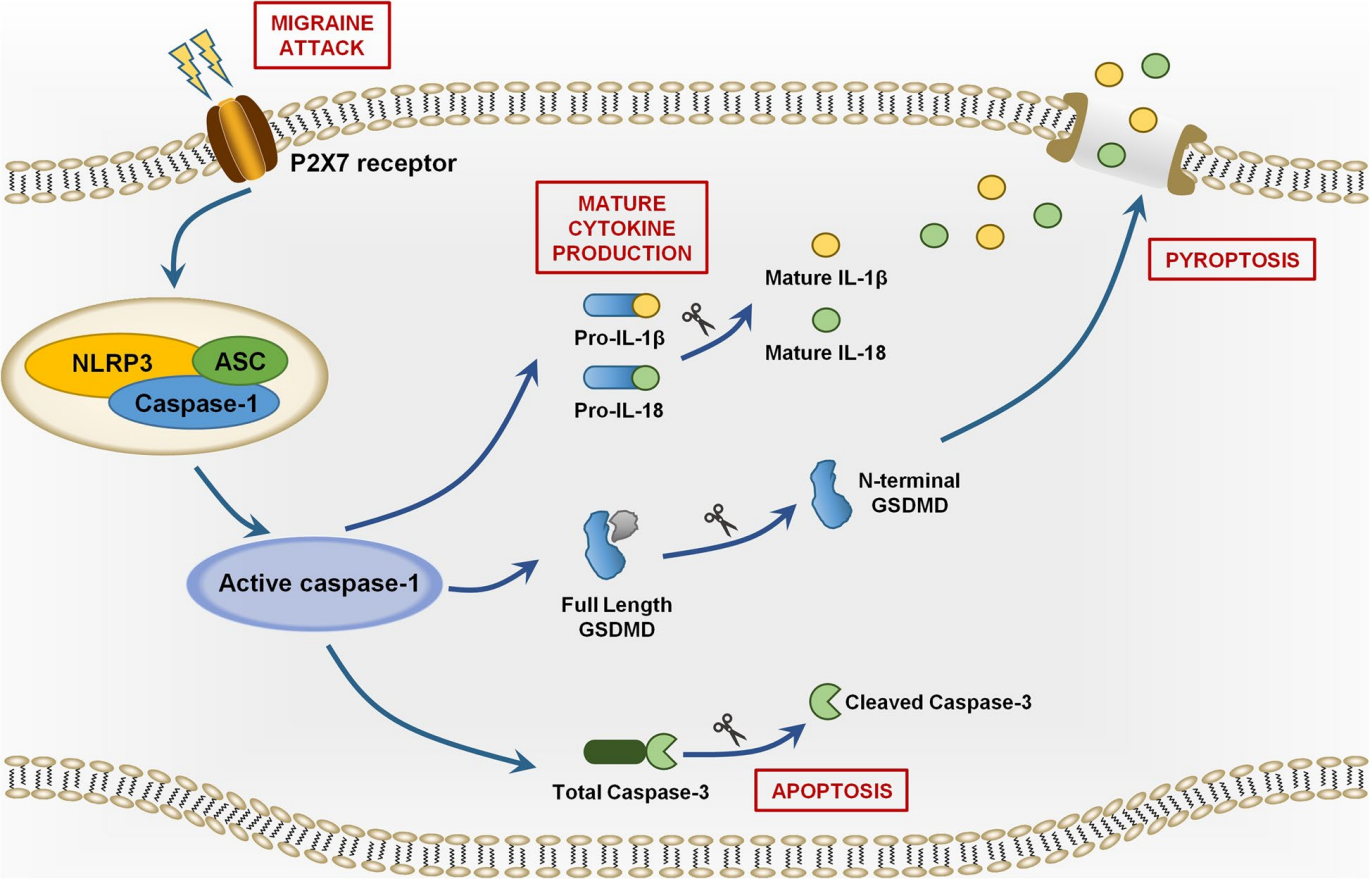
Schematic diagram for the mechanisms of P2X7R in the regulation of inflammasome priming, activation and programmed cell death pathways in the brain following repeated dural IS stimulation. The expression of P2X7R is upregulated after repeated dural IS stimulation. P2X7R can activate the assembly of NLRP3 inflammasome, a multi-protein complex including NLRP3, adaptor (i.e., ASC) and effector proteins (i.e., total caspase-1). The formation of an inflammasome complex activates and converts total caspase-1 into active cleaved caspase-1. There are three main groups of substrates that are targeted by cleaved caspase-1. Firstly, cleaved caspase-1 can cleave both precursor IL-1β and IL-18 into active proinflammatory cytokines, mature IL-1β and IL-18. Secondly, cleaved caspase-1 can cleave GSDMD-FL into GSDMD-NT that self-oligomerize onto the plasma membrane to form a pore to facilitate the influx of water molecules to induce a lytic form of cell death known as pyroptosis. Thirdly, cleaved caspase-1 can cleave and activate total caspase-3 into cleave caspase-3 to induce apoptosis. Abbreviations: GSDMD-FL, full length Gasdermin D; GSDMD-NT, N-terminal Gasdermin D

We further identified downstream signaling pathways following P2X7R activation in the IS-induced migraine model. P2X7R regulates multifaceted cellular signaling pathways including cytokine and chemokine secretion, NLRP3 inflammasome activation, cell death and autophagy [42]. Among them, the P2X7R-NLRP3 signaling pathway mediating inflammatory response and cell death has been shown to be involved in cognitive impairment in multiple neurological diseases such as AD, VaD and diabetes [28–30]. P2X7R activation can initiate the assembly of the NLRP3 inflammasome complex [16, 17], an intracellular multimeric protein complex consisting of a stimulus-detecting sensor NLRP3, the adaptor protein ASC and effector protein total caspase-1 [43]. Stimulation of NLRP3 activates its effector (total caspase-1) into biologically active CC1 [18–20]. The active CC1 facilitates inflammatory response by releasing proinflammatory cytokines IL-1β and IL-18 [21]. The active CC1 may also promote cell death by activating apoptosis and pyroptosis pathways [24, 25]. CC1 induces pyroptosis and apoptosis by cleavage of total caspase-3 and full length GSDMD into CC3 and GSDMD-NT respectively [24, 25]. GSDMD-NT can cause pore formation in the plasma membranes, resulting in leakage of proinflammatory cytokines and cellular content [44, 45]. The P2X7R-NLRP3 signaling pathway was depicted in detail in Fig. 10. It has been shown that NLRP3 inflammasome was activated in the TNC (a central region related to migraine pain) and apoptotic marker (CC3) was increased in the trigeminal ganglia (a peripheral region related to migraine pain) in an NTG-induced migraine mouse model, suggesting that NLRP3 inflammasome activation and apoptosis may be at least partially responsible for migraine pain sensitization (including peripheral and central sensitization) [33, 46]. Our present data indicated that repeated dural IS stimulation led to increased expression of NLRP3 inflammasome components (NLRP3 and total caspase-1), marker of inflammasome activation (CC1), downstream proinflammatory cytokines (IL-1β and IL-18) and pro-pyroptotic protein (GSDMD-NT) in the cerebral cortex (Fig. 3). The pro-apoptotic protein CC3 did not change significantly after dural IS stimulation. The IS-induced NLRP3 inflammasome activation, proinflammatory cytokines release and pyroptosis were attenuated by BBG, a specific P2X7R antagonist (Fig. 5). These findings supported our hypothesis that P2X7R-NLRP3 signaling pathway was activated in the cognition-related brain regions (i.e., cortex and hippocampus) following dural IS stimulation. The present study was the first to demonstrate that NLRP3 inflammasome-mediating pyroptosis occurs in cerebral cortex and hippocampus of a mouse model of IS-induced migraine. This study also provides a possibility that the different ways to induce migraine attacks may activate different cell death pathways in different brain regions and mediate different clinical manifestations of migraine.

### Gliosis and neuronal loss in the cortex and hippocampus following dural IS stimulation

To explore the mechanisms by which the P2X7R-NLRP3 signaling pathway mediates IS-induced cognitive impairment, we examined cognition-related pathological changes including gliosis (microgliosis and astrogliosis) and neuronal loss in the cerebral cortex and hippocampus, and white matter damage. It has been shown that inflammasome activation can mediate cognitive decline by inducing gliosis and neuronal loss in the cerebral cortex and hippocampus, and white matter damage in a mouse model of VaD [27]. Our previous study showed that microgliosis and astrogliosis occurring in the medullary dorsal horn after repeated dural IS stimulation were associated with nociceptive behavior in an animal model of IS-induced migraine.[34]. Here, we found that microgliosis, astrogliosis and neuronal loss were present in the cerebral cortex and hippocampus following dural IS stimulation (Fig. 4), which may account for the structural and functional alterations in the cerebral cortex and hippocampus of migraine patients previously observed in clinical neuroimaging studies [5, 31]. These pathological changes were attenuated by pretreatment with BBG (Figs. 7 and 8), suggesting a role for P2X7R in IS-induced gliosis and neuronal loss.

No obvious white matter lesions were observed in multiple white matter regions following repeated dural IS stimulation (Fig. 4), which was in contrast to four studies using diffusion-weighted magnetic resonance imaging (MRI) [47, 48] and magnetization transfer imaging (MTI) [49, 50], but in line with two MTI studies [32, 51]. A possible explanation might be that migraine patients with white matter lesions were those who had more severe migraine attacks and longer disease duration. Moreover, the MBP and LFB staining used in the present study can only detect visible white matter lesions. Focal invisible microstructural white matter changes might occur in migraine patients preceding visible focal white matter lesions [49, 50]. Thus, our results could not rule out the potential involvement of white matter damage in migraine-related cognitive impairment. Further studies are needed to evaluate the white matter lesions in a more severe migraine model with more sensitive indicators.

### The cellular specificity of inflammasome activation and cell death following dural IS stimulation

To clarify whether inflammasome activation and cell death are cell-specific, we examined the cellular localization of markers of inflammasome activation (CC1), apoptosis (CC3) and pyroptosis (GSDMD). The results indicated that inflammasome CC1 and pro-pyroptotic GSDMD were mainly expressed in neurons and microglia, whereas pro-apoptotic CC3 was mainly expressed in neurons (Fig. 3). The similar cellular localization of CC1 and GSDMD provides evidence for the occurrence of inflammasome-mediating pyroptosis in cortical and hippocampal neurons and microglia in an IS-induced migraine mouse model. The pro-apoptotic CC3 was mainly expressed in cortical and hippocampal neurons, but its expression level was not affected by dural IS stimulation. A possible explanation for this is that the apoptosis in neurons may be induced by cannula implantation surgery, independent of the dural IS stimulation. Despite increased apoptosis was reported in the trigeminal ganglion in an NTG-induced migraine mouse model [46], there is no evidence of apoptosis in central nervous system of migraine patients or animal models of migraine, which is consistent with the present study. No colocalization of CC1, CC3 and GSDMD was observed in astrocytes, indicating inflammasome activation and cell death did not occur in astrocytes, which is in contradiction with astrogliosis following dural IS stimulation. The precise molecular mechanisms for this remain unclear. It is possible that the astrogliosis might be a compensatory mechanism secondary to IS-induced brain injury, and might play a role in neuronal repairment. The number of CC1-positive and GSDMD-positive neurons and microglia decreased after application of BBG (Fig. 6), indicating that the inflammasome activation and pyroptosis in neurons and microglia were mediated by P2X7R.

### Inhibition of P2X7R improved IS-induced cognitive impairment

We further evaluated the effects of P2X7R on nociceptive and cognitive behaviors in the IS-induced migraine model. The role of P2X7R for migraine-related pain symptoms has been elucidated in an NTG-induced migraine mouse model, which reported that inhibition of P2X7R can alleviate NTG-induced mechanical and thermal hyperalgesia [12, 13]. Our results indicated that the reduced periorbital mechanical pain threshold and increased spontaneous pain behavior of the IS mice can be alleviated by inhibition of P2X7R (Fig. 9), supporting the previous findings [52, 53]. The results of cognitive behavior tests showed that the IS mice exhibited a poor recognition memory in novel object recognition test, and impaired spatial memory retention in probe trial of Morris water maze, but no obvious impairment of spatial learning ability in the learning period of Morris water maze (Fig. 9). Inhibition of P2X7R can improve the IS-induced selective cognitive impairment, indicating that P2X7R is a key contributor to migraine-related cognitive impairment. The selective cognitive impairment may be due to the more severe IS-induced brain injury in the cerebral cortex than in the hippocampus. It has been shown that the spatial learning ability in the Morris water maze critically depends on hippocampal function [54], while the recognition memory in novel object recognition test mainly relies on limbic-cortical function [55]. A recent clinical study reported that the impaired visuospatial processing of migraine patients was associated with abnormal recruitment of cortical areas including the anterior insula, medial frontal and orbitofrontal cortex [5], demonstrating a key role of cortical areas in migraine-related cognitive deficits. However, these cognitive behavioral results were in contrast with a previous study conducted on a genetic mouse model of familial hemiplegic migraine type 1 (FHM-1) [56]. This study reported that FHM-1 gain-of-function mutations selectively impaired the spatial memory in Morris water maze by enhancing hippocampal long-term potentiation, but had no effect on non-spatial recognition memory in novel object test [56]. Different animal models of migraine used in the two studies might be a major cause for the contradictory results. Several clinical neuropsychological researches reported that cognitive symptoms in migraine patients are mainly presented as visuospatial abilities, processing speed, attention and executive functions [3–6]. Moreover, neuroimaging studies have revealed that the structural and functional alterations in the cerebral cortex and hippocampus were involved in cognitive impairment in migraine patients [5, 31]. Since any animal models of migraine cannot fully mimic the complex pathogenesis and clinical manifestation of migraine, clinical studies are needed to explore the correspondence between the affected cognitive domains and specific brain regions in migraine patients.

## Limitations

The present study has some limitations that should be noted. Firstly, the downstream signaling pathways of P2X7R may differ in humans and rodents. The findings of the research need to be further verified in patients with migraine. Secondly, the potential off-target effects of BBG may influence the current results, but the consistency of P2X7R downregulation and NLRP3 inflammasome inhibition supports a specific effect of BBG on P2X7R inhibition. We will use P2X7R knockout mice in subsequent studies to provide more convincing evidence for the role of P2X7R in migraine-related cognitive impairment. Thirdly, P2X7R has been shown to regulate multifaceted cellular signaling pathways including cytokine and chemokine secretion, NLRP3 inflammasome activation, cell death and autophagy, some of which were not fully examined in the present study. Fourthly, the contributions of NLRP3 and GSDMD to the migraine-related cognitive impairment need to be further investigated using targeted therapeutic compounds. Fifthly, the IS-induced migraine mouse model used in the present study is unable to completely reproduce cognitive symptoms identical to those seen in migraineurs. More in-depth studies of other established migraine animal models and migraine patients are needed in the future to generalize these findings. Sixthly, the whole cerebral cortex was included in the analysis in this study. Given the different functions of different cortical regions, it is necessary to explore the specific roles of different cortical regions in migraine-related cognitive impairment in the future.

## Conclusions

In summary, this study demonstrated the role of P2X7R-NLRP3 signaling pathway in promoting neuroinflammation, pyroptosis, gliosis and neuronal loss induced by dural IS stimulation. Inhibition of P2X7R using BBG mitigated these IS-induced brain injury and improved cognitive impairment in the IS-induced migraine mouse model. Our results have identified P2X7R as a key contributor to migraine-related cognitive impairment.

## Supplementary Information

**Additional file 1:** **SupplementaryFig. 1. **Effect of repeated duralIS stimulation on the cellular specificity of inflammasome-mediated programmedcell death in the cerebral cortex and hippocampus. (a-f)Double immunofluorescence staining showed that cleaved caspase-1 (CC1) andGSDMD were co-localized within neurons (NeuN positive) and microglia (Iba-1positive) in the cerebral cortex (a, c) and hippocampus (d, f), while cleavedcaspase-3 (CC3) was co-localized within neurons (NeuN positive) (b, e). Zoom magnification × 40.Scale bar, 20 μm. Images were taken under identical exposures and conditions.The regions boxed with white dashed lines were shown in Fig. 3f-g. Abbreviations:IS, inflammatory soup; CC1, cleaved caspase-1; CC3, cleaved caspase-3; GSDMD,Gasdermin D; NeuN, neuronal nuclei; Iba-1, ionized calcium-binding adaptormolecule-1; GFAP, glial fibrillary acidic protein

**Additional file 2:** **SupplementaryFig. 2. **Effect of repeated duralIS stimulation on inflammasome activation in multiple cell types in thecerebral cortex and hippocampus. (a-f) Representativeindividual immunofluorescence images of DAPI (nucleus marker), cleavedcaspase-1 (inflammasome activation marker) and neuronal (NeuN positive),microglial (Iba-1 positive) and astroglia (GFAP positive) immunoreactivity inthe cerebral cortex and hippocampus of sham mice and IS mice. Magnification x40. Scale bar, 20 μm. Images were taken under identical exposures andconditions. Abbreviations: IS,inflammatory soup; NeuN, neuronal nuclei; Iba-1, ionized calcium-bindingadaptor molecule-1; GFAP, glial fibrillary acidic protein.

**Additional file 3:** **SupplementaryFig. 3. **Effect of repeated dural IS stimulation on apoptoticcell death in multiple cell types in the cerebral cortex and hippocampus. (a-f) Representativeindividual immunofluorescence images of DAPI (nucleus marker), cleavedcaspase-3 (apoptosis marker) and neuronal (NeuN positive), microglial (Iba-1positive) and astroglia (GFAP positive) immunoreactivity in the cerebral cortexand hippocampus of sham mice and IS mice. Magnification x 40. Scale bar, 20 μm.Images were taken under identical exposures and conditions. Abbreviations: IS,inflammatory soup; NeuN, neuronal nuclei; Iba-1, ionized calcium-bindingadaptor molecule-1; GFAP, glial fibrillary acidic protein.

**Additional file 4:** **SupplementaryFig. 4. **Effect of repeated dural IS stimulation onpyroptotic cell death in multiple cell types in the cerebral cortex andhippocampus. (a-f)Representative individual immunofluorescence images of DAPI (nucleus marker),GSDMD (pyroptosis marker) and neuronal (NeuN positive), microglial (Iba-1positive) and astroglia (GFAP positive) immunoreactivity in the cerebral cortexand hippocampus of sham mice and IS mice. Magnification x 40. Scale bar, 20 μm.Images were taken under identical exposures and conditions. Abbreviations: IS,inflammatory soup; NeuN, neuronal nuclei; Iba-1, ionized calcium-bindingadaptor molecule-1; GFAP, glial fibrillary acidic protein.

**Additional file 5:** **SupplementaryFig. 5. **Effect of inhibition ofP2X7R on the cellular specificity of inflammasome-mediated programmed celldeath in the cerebral cortex and hippocampus following repeated dural ISstimulation. (a, d) Double immunofluorescence staining showed areduction in CC1-positive (marker of inflammasome activation)neurons (NeuN positive) and microglia (Iba-1 positive) in the cerebral cortexand hippocampus of IS-BBG mice compared to IS-VEH mice.(b, e) Doubleimmunofluorescence showed similar CC3-positive (apoptotic marker) neurons (NeuNpositive) in the cerebral cortex and hippocampus among the four groups. (c, f)Double immunofluorescence staining showed a reduction in GSDMD-positive(pyroptotic marker) neurons (NeuN positive) and microglia (Iba-1 positive) inthe cerebral cortex and hippocampus of IS-BBG mice compared to IS-VEH mice. Magnificationx 40. Scale bar, 20 μm. Images were taken under identical exposures andconditions. The regions boxed with white dashed lines were shown in Fig. 6a-f. Abbreviations:IS, inflammatory soup; CC1, cleaved caspase-1; CC3, cleaved caspase-3; GSDMD,Gasdermin D; NeuN, neuronal nuclei; Iba-1, ionized calcium-binding adaptormolecule-1; GFAP, glial fibrillary acidic protein; BBG, brilliant blue G; VEH,vehicle.

**Additional file 6:** **SupplementaryFig. 6. **Effect of inhibition of P2X7R on inflammasomeactivation in multiple cell types in the cerebral cortex and hippocampusfollowing repeated dural IS stimulation. (a-f) Representative individual immunofluorescenceimages of DAPI (nucleus marker), cleaved caspase-1 (inflammasome activationmarker) and neuronal (NeuN positive), microglial (Iba-1 positive) and astroglia(GFAP positive) immunoreactivity in the cerebral cortex and hippocampus of fourgroups including sham-VEH group, sham-BBG group, IS-VEH group and IS-BBG group.Magnification x 40. Scale bar, 20 μm. Images were taken under identicalexposures and conditions. Abbreviations:IS, inflammatory soup; NeuN, neuronal nuclei; Iba-1, ionized calcium-binding adaptormolecule-1; GFAP, glial fibrillary acidic protein; BBG, brilliant blue G; VEH,vehicle.

**Additional file 7:** **SupplementaryFig. 7. **Effect of inhibition ofP2X7R on apoptotic cell death in multiple cell types in the cerebral cortex andhippocampus following repeated dural IS stimulation.(a-f) Representative individual immunofluorescence images of DAPI (nucleusmarker), cleaved caspase-3 (apoptosis marker) and neuronal (NeuN positive),microglial (Iba-1 positive) and astroglia (GFAP positive) immunoreactivity inthe cerebral cortex and hippocampus of four groups including sham-VEH group,sham-BBG group, IS-VEH group and IS-BBG group. Magnification x 40. Scale bar,20 μm. Images were taken under identical exposures and conditions.Abbreviations: IS,inflammatory soup; NeuN, neuronal nuclei; Iba-1, ionized calcium-bindingadaptor molecule-1; GFAP, glial fibrillary acidic protein; BBG, brilliant blueG; VEH, vehicle.

**Additional file 8:** **SupplementaryFig. 8. **Effect of inhibition of P2X7R on pyroptotic celldeath in multiple cell types in the cerebral cortex and hippocampus followingrepeated dural IS stimulation. (a-f) Representative individual immunofluorescence images of DAPI(nucleus marker), GSDMD (pyroptosis marker) and neuronal (NeuN positive),microglial (Iba-1 positive) and astroglia (GFAP positive) immunoreactivity inthe cerebral cortex and hippocampus of four groups including sham-VEH group,sham-BBG group, IS-VEH group and IS-BBG group. Magnification x 40. Scale bar,20 μm. Images were taken under identical exposures and conditions. Abbreviations: IS, inflammatory soup;NeuN, neuronal nuclei; Iba-1, ionized calcium-binding adaptor molecule-1; GFAP,glial fibrillary acidic protein; BBG, brilliant blue G; VEH, vehicle.

## Data Availability

All data generated or analysed during this study are included in this article.

## Abbreviations

AD: Alzheimer’s disease
ANOVA: Analysis of variance
BBG: Brilliant Blue G
BSA: Bovine serum albumin
CC1: Cleaved caspase-1
CC3: Cleaved caspase-3
CO_2_: Carbon dioxide
CSD: Cortical spreading depression
DG: Dentate gyrus
DS: Dural stimulation
ELISA: Enzyme-linked immunosorbent assay
FHM-1: Familial hemiplegic migraine type 1
GFAP: Glial fibrillary acidic protein
GSDMD-FL: Full length Gasdermin D
GSDMD-NT: N-terminal GSDMD
HRP: Horseradish peroxidase
IACUC: Institutional Animal Care and Use Committee
Iba-1: Ionized calcium-binding adaptor molecule-1
i.p.: Intraperitoneal injection
IS: Inflammatory soup
LFB: Luxol-fast-blue
MAP2: Microtubule-associated protein 2
MBP: Myelin basic protein
MRI: Magnetic resonance imaging
MTI: Magnetization transfer imaging
NeuN: Neuronal nuclei
NTG: Nitroglycerin
P2X7R: P2X7 receptor
PBS: Phosphate buffer saline
PVDF: Polyvinylidene-difluoride
SDS-PAGE: Sulfate–polyacrylamide gel electrophoresis
SEM: Standard error of the mean
SPF: Specific pathogen-free
TNC: Trigeminal nucleus caudalis
T_novel_: Time spent exploring novel object
T_old_: Time spent exploring old object
VaD: Vascular dementia
VEH: Vehicle
VFT: Von Frey’s test
