# Supplementary figures and images for: P2X7R/NLRP3 signaling pathway-mediated pyroptosis and neuroinflammation contributed to cognitive impairment in a mouse model of migraine
Source: J Headache Pain. 2022 Jul 2;23(1):75. doi: 10.1186/s10194-022-01442-8 (PMC9250730; doi:10.1186/s10194-022-01442-8)

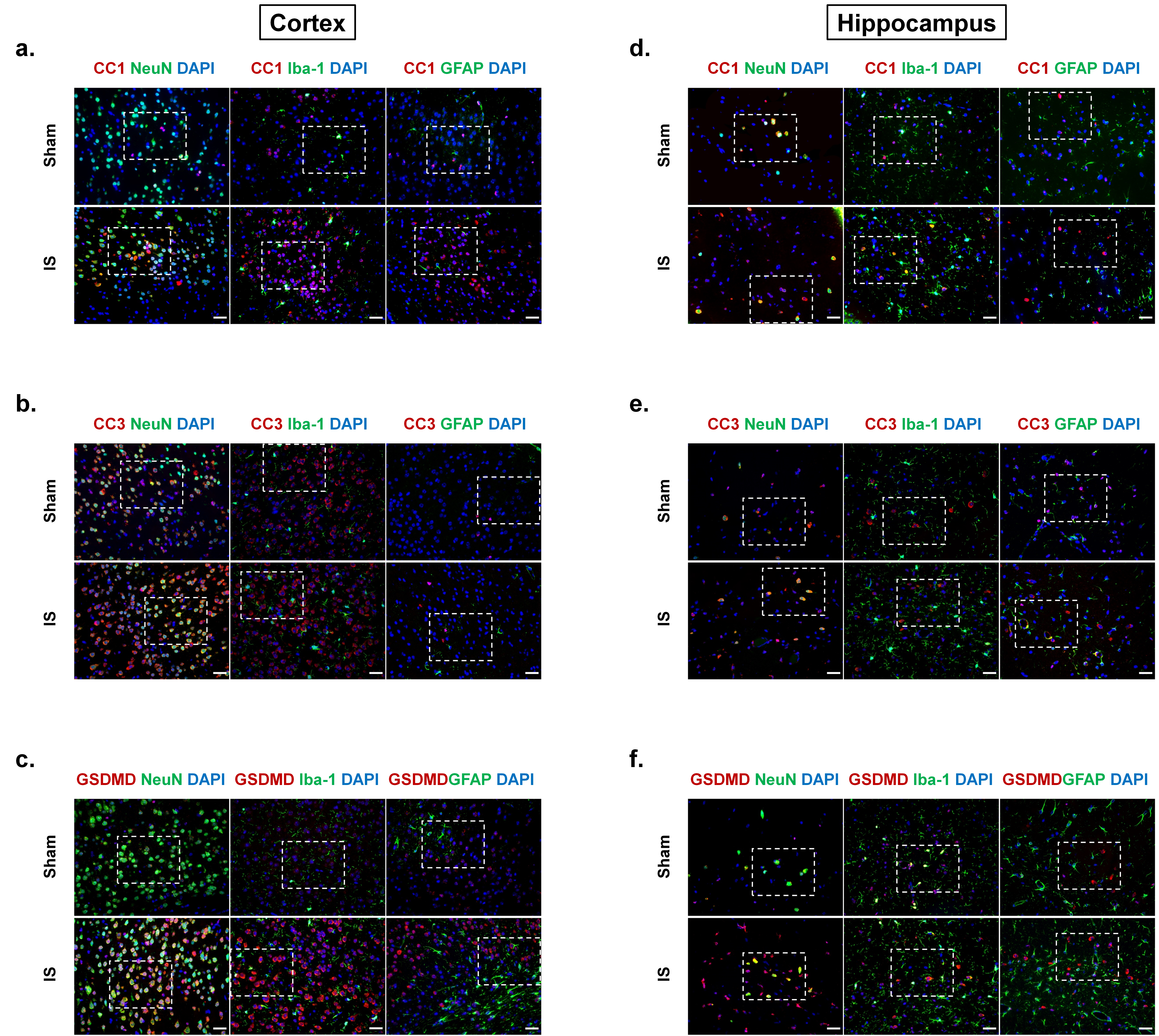

Supplement: Supplementary file 1 — Additional file 1: SupplementaryFig. 1. Effect of repeated duralIS stimulation on the cellular specificity of inflammasome-mediated programmedcell death in the cerebral cortex and hippocampus. (a-f)Double immunofluorescence staining showed that cleaved caspase-1 (CC1) andGSDMD were co-localized within neurons (NeuN positive) and microglia (Iba-1positive) in the cerebral cortex (a, c) and hippocampus (d, f), while cleavedcaspase-3 (CC3) was co-localized within neurons (NeuN positive) (b, e). Zoom magnification × 40.Scale bar, 20 μm. Images were taken under identical exposures and conditions.The regions boxed with white dashed lines were shown in Fig. 3f-g. Abbreviations:IS, inflammatory soup; CC1, cleaved caspase-1; CC3, cleaved caspase-3; GSDMD,Gasdermin D; NeuN, neuronal nuclei; Iba-1, ionized calcium-binding adaptormolecule-1; GFAP, glial fibrillary acidic protein [file 10194_2022_1442_MOESM1_ESM.tif]

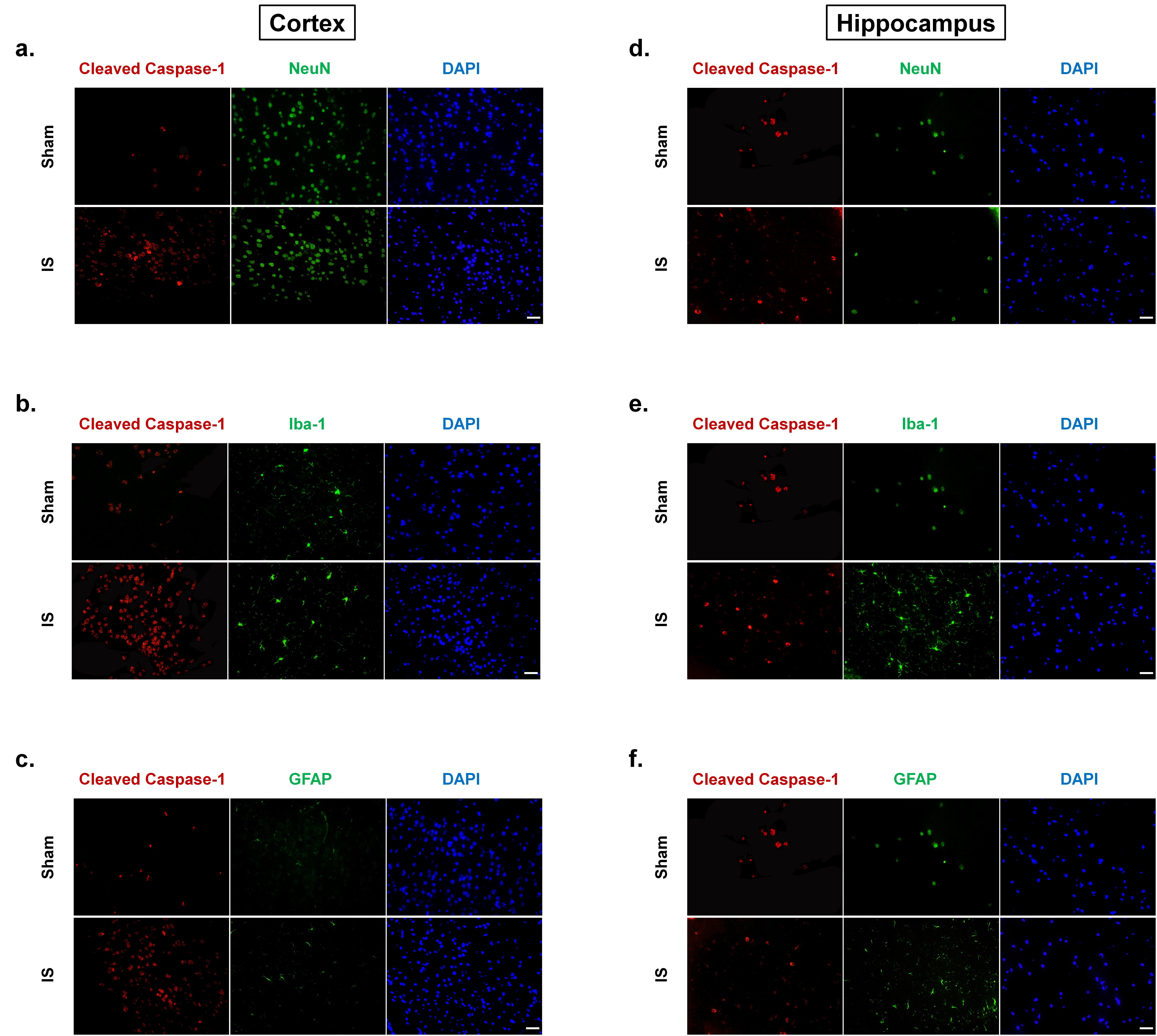

Supplement: Supplementary file 2 — Additional file 2: SupplementaryFig. 2. Effect of repeated duralIS stimulation on inflammasome activation in multiple cell types in thecerebral cortex and hippocampus. (a-f) Representativeindividual immunofluorescence images of DAPI (nucleus marker), cleavedcaspase-1 (inflammasome activation marker) and neuronal (NeuN positive),microglial (Iba-1 positive) and astroglia (GFAP positive) immunoreactivity inthe cerebral cortex and hippocampus of sham mice and IS mice. Magnification x40. Scale bar, 20 μm. Images were taken under identical exposures andconditions. Abbreviations: IS,inflammatory soup; NeuN, neuronal nuclei; Iba-1, ionized calcium-bindingadaptor molecule-1; GFAP, glial fibrillary acidic protein. [file 10194_2022_1442_MOESM2_ESM.tif]

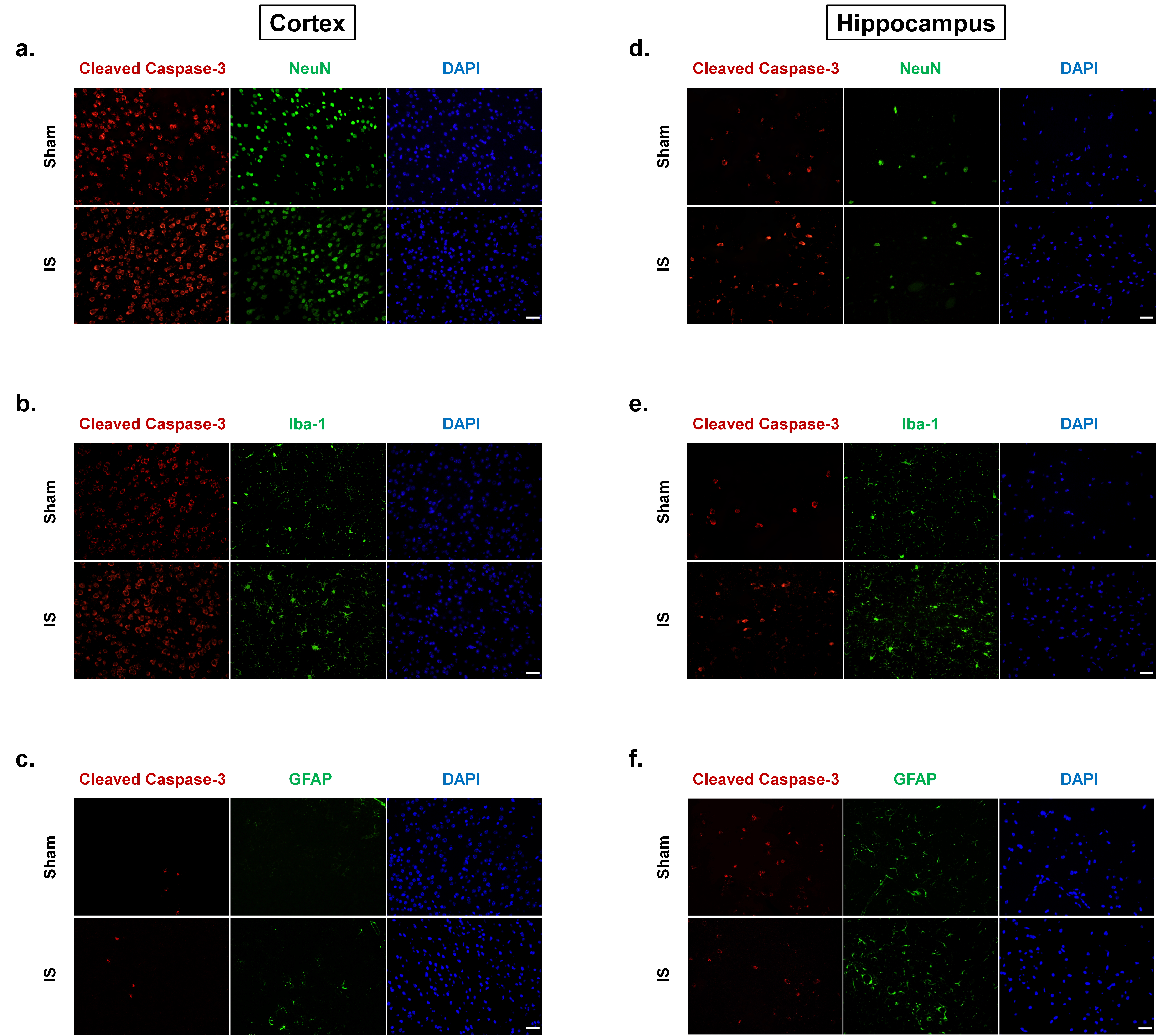

Supplement: Supplementary file 3 — Additional file 3: SupplementaryFig. 3. Effect of repeated dural IS stimulation on apoptoticcell death in multiple cell types in the cerebral cortex and hippocampus. (a-f) Representativeindividual immunofluorescence images of DAPI (nucleus marker), cleavedcaspase-3 (apoptosis marker) and neuronal (NeuN positive), microglial (Iba-1positive) and astroglia (GFAP positive) immunoreactivity in the cerebral cortexand hippocampus of sham mice and IS mice. Magnification x 40. Scale bar, 20 μm.Images were taken under identical exposures and conditions. Abbreviations: IS,inflammatory soup; NeuN, neuronal nuclei; Iba-1, ionized calcium-bindingadaptor molecule-1; GFAP, glial fibrillary acidic protein. [file 10194_2022_1442_MOESM3_ESM.tif]

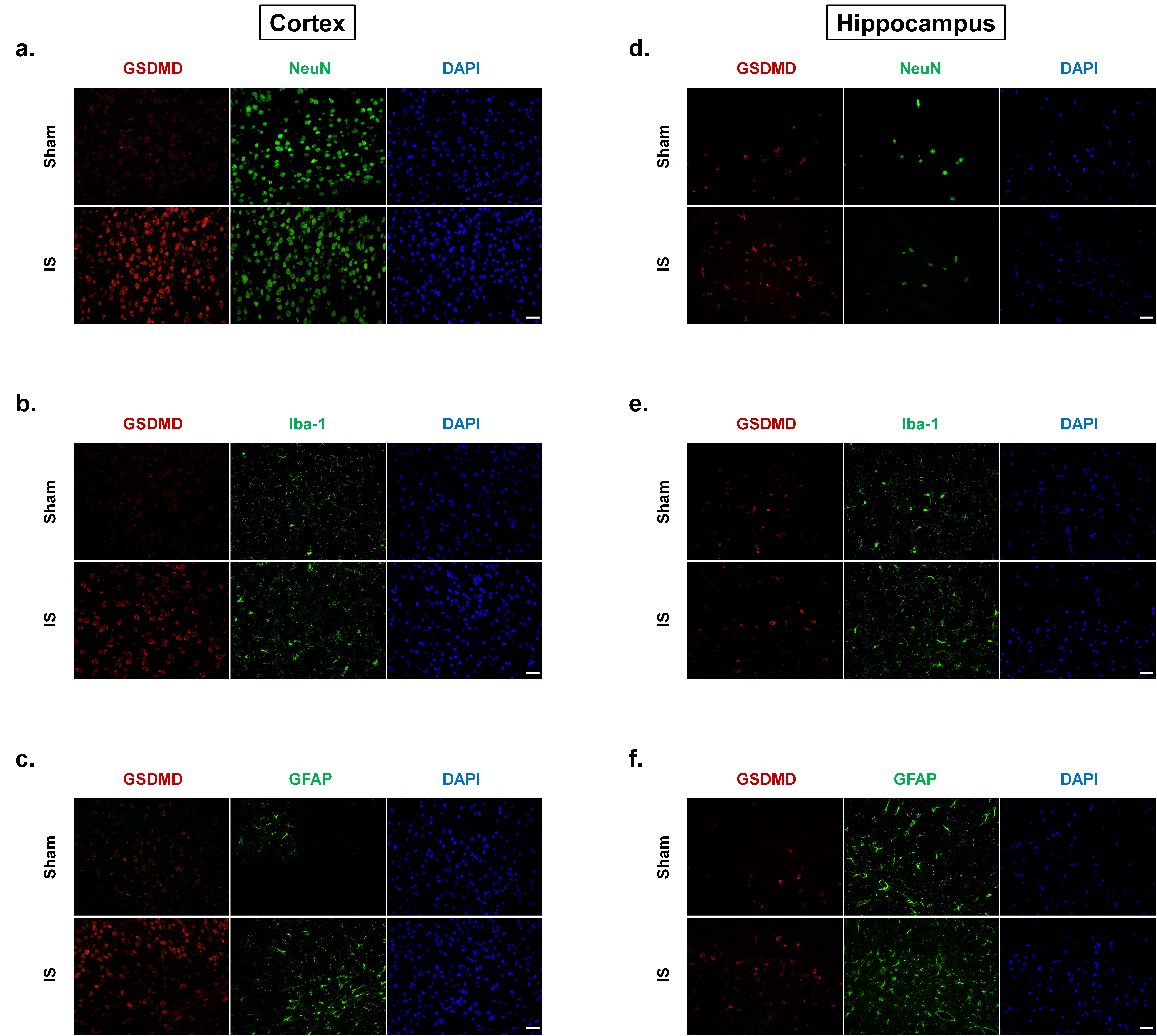

Supplement: Supplementary file 4 — Additional file 4: SupplementaryFig. 4. Effect of repeated dural IS stimulation onpyroptotic cell death in multiple cell types in the cerebral cortex andhippocampus. (a-f)Representative individual immunofluorescence images of DAPI (nucleus marker),GSDMD (pyroptosis marker) and neuronal (NeuN positive), microglial (Iba-1positive) and astroglia (GFAP positive) immunoreactivity in the cerebral cortexand hippocampus of sham mice and IS mice. Magnification x 40. Scale bar, 20 μm.Images were taken under identical exposures and conditions. Abbreviations: IS,inflammatory soup; NeuN, neuronal nuclei; Iba-1, ionized calcium-bindingadaptor molecule-1; GFAP, glial fibrillary acidic protein. [file 10194_2022_1442_MOESM4_ESM.tif]
